# Up right, not right up: Primacy of verticality in both language and movement

**DOI:** 10.3389/fnhum.2022.981330

**Published:** 2022-09-29

**Authors:** Véronique Boulenger, Livio Finos, Eric Koun, Roméo Salemme, Clément Desoche, Alice C. Roy

**Affiliations:** ^1^Laboratoire Dynamique Du Langage, UMR 5596, CNRS/University Lyon 2, Lyon, France; ^2^Department of Statistical Sciences, University of Padua, Padua, Italy; ^3^Integrative Multisensory Perception Action & Cognition Team (IMPACT), Lyon Neuroscience Research Center, INSERM U1028, CNRS U5292, University Lyon 1, Lyon, France; ^4^Neuro-Immersion, Lyon Neuroscience Research Center, Lyon, France

**Keywords:** verticality, hand pointing movement, language, semantic typology, kinematics

## Abstract

When describing motion along both the horizontal and vertical axes, languages from different families express the elements encoding verticality before those coding for horizontality (e.g., *going up right* instead of *right up*). In light of the motor grounding of language, the present study investigated whether the prevalence of verticality in Path expression also governs the trajectory of arm biological movements. Using a 3D virtual-reality setting, we tracked the kinematics of hand pointing movements in five spatial directions, two of which implied the vertical and horizontal vectors equally (i.e., up right +45° and bottom right −45°). Movement onset could be prompted by visual or auditory verbal cues, the latter being canonical in French (“en haut à droite”/up right) or not (“à droite en haut”/right up). In two experiments, analyses of the index finger kinematics revealed a significant effect of gravity, with earlier acceleration, velocity, and deceleration peaks for upward (+45°) than downward (−45°) movements, irrespective of the instructions. Remarkably, confirming the linguistic observations, we found that vertical kinematic parameters occurred earlier than horizontal ones for upward movements, both for visual and congruent verbal cues. Non-canonical verbal instructions significantly affected this temporal dynamic: for upward movements, the horizontal and vertical components temporally aligned, while they reversed for downward movements where the kinematics of the vertical axis was delayed with respect to that of the horizontal one. This temporal dynamic is so deeply anchored that non-canonical verbal instructions allowed for horizontality to precede verticality only for movements that do not fight against gravity. Altogether, our findings provide new insights into the embodiment of language by revealing that linguistic path may reflect the organization of biological movements, giving priority to the vertical axis.

## Introduction

English and French speakers can “climb the stairs backwards” and “monter les escaliers à reculons,” or “click on the top right corner of a screen” and “cliquer en haut à droite d’un écran.” These instances express Path, a research topic that has been extensively studied in cognitive linguistics and semantic typology. Path refers to the place occupied or the path followed by an entity (i.e., the Figure) with respect to a reference entity (i.e., the Ground; Talmy, 1985, 1991, 2000; see also Imbert, 2012). Path can be characterized by its Axiality, namely whether it is organized with respect to a vertical (e.g., *up* and *down*) or horizontal (e.g., *backward* and *forward*) axis (Imbert, 2013). In the above examples, Path is expressed both on the vertical and horizontal axes and it is noteworthy that the elements encoding verticality are expressed first, within or closer to the verb, before those elements encoding horizontal Path. It would indeed seem odd to “go back the stairs up.” Investigations in linguistic typology suggest that these instances may actually reflect a tendency of several languages to favor the vertical direction over the horizontal one when expressing Path. In a crosslinguistic comparison, Imbert (2013, 2016) reported that languages from different language families (from Mayan to Sinitic languages through Indo-European languages) that otherwise share little regarding their morphosyntactic rules show striking similarities in organizing axial Path-encoding elements. When the Figure moves both along the vertical and horizontal axes, the morphemes encoding vertical Path are always closer to the main verb or verb root (i.e., they are encoded first) than the elements encoding horizontal Path. Many languages also demonstrate an asymmetry between their multiple ways of expressing verticality (e.g., above/over, upward, higher…) and the paucity of words expressing locations on the horizontal and sagittal axes (e.g., to the left/right, in front of/behind) (Levinson, 2003, p. 46; see also Forker, 2020 for an asymmetry in demonstratives). Besides, constant spatial relations between objects along the vertical axis contrast with the changing relations on the horizontal plane. This asymmetry may explain why, after reading narratives, participants are faster to judge the spatial position of objects with respect to a reference object when they are located on the vertical rather than the horizontal axis (Bryant et al., 1992).

These typological findings raise the question of whether the primacy of verticality in language may find an echo in other related domains such as biological movement. Large empirical evidence reveals that language and action do not only co-exist but share functional commonalities. The most obvious instantiations of this interplay are probably co-verbal manual gestures. Gestures spontaneously accompany speech irrespective of the culture and linguistic background, even in congenitally blind speakers (Iverson and Goldin-Meadow, 1998), and would ease lexical access and word retrieval in children with language impairment (Iverson and Braddock, 2011) and patients with aphasia (Hadar et al., 1998; Lanyon and Rose, 2009; see also Krauss et al., 2000). Healthy gesturing speakers have been shown to omit necessary spatial information in their verbal descriptions of pictures more often than non-gesturing speakers (Melinger and Levelt, 2004; see also Graham and Heywood, 1975 for more elaborated verbal expressions in speakers who were not allowed to gesture). The existence of co-verbal gestures in every culture and language has bolstered the hypothesis that language evolved from manual gestures (Arbib, 2002; Corballis, 2002, 2010; Gentilucci and Corballis, 2006). Convincing evidence has shown that language and manual actions indeed entertain a close relationship. Developmental studies revealed that fine and gross motor skills are predictive markers of concurrent and subsequent language development in infancy and childhood (Bates and Dick, 2002; Iverson, 2010; Gonzalez et al., 2019). The onset of reduplicated babbling coincides with increased rhythmic arm movements (Locke et al., 1995; Iverson et al., 2007). The use of deictic gestures toward objects and of gesture-plus-word combinations furthermore predicts the production of words and of two-word combinations, respectively (Iverson and Goldin-Meadow, 2005; Iverson et al., 2008). Motor and language development therefore go hand in hand, which may account for the frequent co-occurrence of motor and language disorders in atypical development (Hill, 2001).

Studies in adults also highlight the intimate links between language and manual actions. Hand and mouth motor representations occupy close cortical territories (Farnè et al., 2002) and most noticeably, speech perception and production do not only increase the excitability of the oro-facial motor cortical region (Roy et al., 2008; D’Ausilio et al., 2012) but also that of the hand motor representation (Flöel et al., 2003; Meister et al., 2007). Nice parallels between syllable production and execution/observation of arm movements have also been reported (Higginbotham et al., 2008; Gentilucci et al., 2009; see also Bernardis et al., 2008 for evidence in 9- to 11-month-old infants). Grasping a large object, with respect to a smaller one, while pronouncing the syllable /ba/ induces larger lip opening and increases the vowel second formant (Gentilucci, 2003; Gentilucci et al., 2004a,b). Reciprocally, finger opening is larger when participants simultaneously articulate the open vowel /a/ as opposed to closed vowel /i/ (Gentilucci and Campione, 2011). Other studies furthermore revealed systematic correspondence between grip types and articulatory gestures (Vainio et al., 2013, 2014, 2017; Tiainen et al., 2017). Beyond speech perception and production, a large body of behavioral and neuroimaging studies has also demonstrated that semantic processing of language is grounded in the sensorimotor system (see Willems and Hagoort, 2007; Fischer and Zwaan, 2008; Pulvermüller and Fadiga, 2010 for reviews; see also Roy et al., 2013; Brozzoli et al., 2019; Thibault et al., 2021 for evidence for syntax). The motor cortex resonates somatotopically during processing of words or sentences describing bodily actions (Hauk et al., 2004; Tettamanti et al., 2005; Boulenger et al., 2009; Desai et al., 2010) and in turn, action words can affect the kinematics of movement execution (Boulenger et al., 2006; Fargier et al., 2012; see also Frak et al., 2010). Compatibility effects between the direction evoked by sentences and the direction of manual responses have also been reported (Glenberg and Kaschak, 2002; Gianelli et al., 2011; Aravena et al., 2012). Interestingly, words have also been shown to modulate low-level sensory perception. In a recent study, we revealed the facilitatory influence of reading verbs denoting tactile perception on the speed of detection of tactile stimulations (Boulenger et al., 2020). In the visual domain, Meteyard et al. (2007) nicely demonstrated that upward and downward motion verbs reduced perceptual sensitivity for the detection of incongruent vertical motion (see also Richardson et al., 2003; Zwaan et al., 2004; Kaschak et al., 2005; Meteyard et al., 2008). Verticality is central to perception – of our body configuration and of our environment – and to action, especially in relation to gravity. Indeed, knowing which way is up or down is fundamental to handle gravitational forces and thus maintain verticality for posture and safe locomotion. When performing upward or downward movements toward objects, we also excel in continuously controlling for gravity loads of our upper limb and of the objects for optimal motor execution (White et al., 2020 for a review).

Given the intertwining of language and action, we here hypothesized that the same rules may govern the trajectory of arm movements and the expression of Path in language, namely by prioritizing the vertical axis over the horizontal one. To test this, we tracked the kinematics of arm pointing movements in five different spatial directions in a 3D virtual-reality setting. Crucially for our purpose, targets to be pointed could be located up right or bottom right in the virtual environment, namely they implied equal horizontal and vertical vectors. Through fine-grained analysis of the finger kinematics in the X, Y, Z coordinate frame, we assessed whether parameters such as acceleration and velocity peaks occur earlier on the vertical (Z) than on the horizontal axis (X) when movements imply both directions. In one block, visual (non-verbal) cues indicated the targets to point, allowing to test the primacy of verticality in biological movements irrespective of language. In another block, auditory verbal instructions were provided to assess the potential imprint of language on action. Verbal instructions could be either congruent (“en haut à droite”/“up right”) or incongruent (“à droite en haut”/“right up”) with the organization of Path-encoding elements in French. We examined whether and how non-canonical linguistic Path expression that do not prioritize verticality affects movement kinematics with respect to canonical instructions.

## Experiment 1

### Materials and methods

#### Participants

Twenty French native healthy adults [13 females, mean age ± SD = 20.4 ± 1.76 years old, age range (18–24 years old)] took part in this experiment. All were right-handed according to the Edinburgh handedness inventory (Oldfield, 1971) and had normal or corrected-to-normal vision. They reported no language, motor or any other neurological disorders. Participants were naive to the purpose of the study. The protocol conformed to the declaration of Helsinki and was approved by a national ethics committee (Comité de Protection des Personnes Sud-Est II). All participants signed an informed consent before the experiment and they were paid for their participation.

#### Apparatus and stimuli

The experiment took place at the Neuro-immersion research facility,^1^ using virtual reality (VR) and a VICON^®^ optical-passive motion capture system. VICON^®^ uses cameras to track the position of reflective markers illuminated by near infrared (NIR) light source with a submillimeter accuracy (between 0.06 and 0.15 mm in static condition, and 0.2/0.3 mm in dynamic condition; Windolf et al., 2008; Merriaux et al., 2017). We used a setup of 7 camera Bonita (1-megapixel resolution) with the software Vicon Tracker^®^, that allowed to acquire participants’ motion with a frequency of 250 Hz. Participants were equipped with a virtual-reality headset (Oculus Rift^2^; resolution: 960 × 1,080 per eye, frequency: 75 Hz, field of view: 106°). Two reflective markers were placed on their right arm: one on their wrist and one on the tip of their index finger. A third marker was placed on their right shoulder for calibration (see Procedure). The experiment was implemented within Unity (Version 5.2.2; Unity Technologies, San Francisco, CA, USA) and Oculus Runtime (Oculus Configuration Utility version 1.10, SDK 0.8.0.0) software. These were used to create the VR environment with an avatar, display experimental stimuli on the head-mounted display (HMD) and through a loudspeaker (for verbal instructions), and record the exact position of all tracked elements (markers on the wrist and index finger). The experiment was run on a computer with an Intel Core i7 processor, Nvidia 1080 8G graphics card, and Windows 10 operating system. The scene was rendered in Oculus Rift DK2 software (Oculus Configuration Utility version 1.10, SDK 0.8.0.0).

**FIGURE 1:**
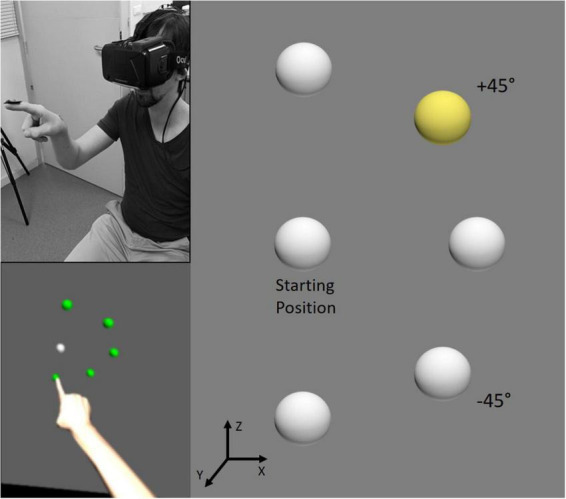
Illustration of the experimental setup. The participant was equipped with an Oculus Rift and was required to point to one of five spherical targets in the virtual environment. All targets were equidistant from the starting position and located up, up right (+45°), right, bottom right (–45°) or at the bottom. In the visual block, the target to point was indicated by a change of color of the sphere from gray to yellow (as illustrated on the right panel for the +45° target), whereas in the verbal block the instruction was delivered auditorily. Feedback was provided by coloring all spheres in green (see the bottom left panel for another trial) for correct pointing, and in red for incorrect pointing.

Targets for pointing movements were five virtual 3D light-gray spheres (diameter 25 mm) located on the right part of a virtual plane situated in front of the participants, 45 cm from their sternum. These spherical targets were equidistant from 15 cm from a virtual central starting point (sphere of the same diameter) also located in front of the participants at the level of their chin. In the X, Y, Z coordinate frame, targets were displayed at five different spatial positions with respect to the horizontal axis (X): 90° (up), +45° (up right), 0° (right), −45° (bottom right), and bottom (−90°; Figure 1).

The experiment was divided in two blocks. In one block, the instructions on which target to point were visual, namely a change of color of the target. In another block, verbal instructions were delivered auditorily. There was a total of seven verbal instructions. Three of them were recorded (44.1 kHz, mono, 16 bits) by a male French native speaker in a sound-attenuated booth using *ROCme* software (Ferragne et al., 2012): “en haut” (up; stimulus duration = 340 ms), “à droite” (right; 471 ms) and “en bas” (bottom; 396 ms). The four remaining instructions, “en haut à droite” (up right), “en bas à droite” (bottom right), “à droite en haut” (right up) and “à droite en bas” (right bottom), were created by concatenating the three previously recorded stimuli using Praat (Boersma and Weenik, 2012). This prevented any effect of stimulus duration or pronunciation (e.g., intonation) in the comparison between canonical (“en haut à droite” and “en bas à droite”) and non-canonical (“à droite en haut” and “à droite en bas”) expression of Path in French. All sound files were finally normalized at a mean intensity of 70 dB with Praat.

#### Procedure

The participants were sitting on a chair in the experimental room and were equipped with the Oculus Rift. A calibration phase first aimed at verifying that the avatar (female or male depending on the participant) was well-aligned with the markers placed on the participant’s wrist and shoulder as well as adjusted to their height. To determine the arm length of the avatar, participants were asked to point to a position in front of them, 45 cm distant from their chest. The orientation of the avatar’s hand was determined based on the marker on the participant’s index finger. Participants were then asked to point to the central starting position and to each of the five targets sequentially, ensuring all were at a reachable distance. This allowed the collection of the 3D position of the targets (based on the index finger marker). The experiment could then start. Half of the participants started with the visual block and the other half with the verbal block. For each block, written instructions were displayed through the VR headset.

In the visual block, each trial ran as follows: participants were asked to point to the central starting position, which triggered the display of a yellow circular gauge (with no ticks). Participants were required to stay in this position until the gauge swept from 0° to 360° for a duration of 2 s, after which the kinematic recordings started. After a variable delay ranging between 700 and 1000 ms, one of the five spherical targets changed color from gray to yellow, indicating the participants to point to it with their right index finger as fast and accurately as possible. The kinematic recordings ended once the participants reached the target. For a movement to be considered as correct, the participants had to point to the instructed target within 2 s from the go signal (change of color of the target), in a 50 mm zone around the target center and stay on the target for at least 500 ms. Anticipated movements (i.e., movement onset before the go signal), wrong targets, targets not reached within the allocated time of 2 s and targets reached but without maintaining the pointing for the required 500 ms were considered as incorrect trials. Feedback was provided by coloring all spheres in green (for 500 ms) for correct pointing, and in red for incorrect pointing. All spheres then became gray again, indicating that the next trial could begin. Note that each trial only started once the participants were in the starting position, thus allowing them to rest if needed. Each sphere (in each of the five spatial positions) was presented as a target 15 times, resulting in a total of 75 trials for this visual block. The trials were pseudo-randomized, with no more than two consecutive trials with a target at the same location. Two breaks were proposed to the participants (with equiprobable targets in each of the three sub-blocks).

The procedure for the verbal block was globally similar. After the participants pointed to the central starting position and the circular gauge ended, a verbal instruction was delivered after a variable delay (700 to 1000 ms) through a loudspeaker (mini speaker model JBL GO Portable, HARMAN International Industries, Northridge, CA, USA) placed on a table in front of the participants. The offset of the sound file triggered an immediate change of color of the starting point from gray to yellow, indicating the participants to point toward the instructed target as fast and accurately as possible. Participants were allowed a maximum of 5 s to point to the target, the remaining procedure was similar to that of the visual block. Each of the seven verbal instructions was delivered 15 times in a pseudo-randomized order, for a total of 105 trials.

Trials for which participants anticipated the movements, reached the correct targets but did not validate them (by not pointing to them for at least 500 ms), or did not reach any target within the 2 or 5 s period (for visual and verbal blocks, respectively) were reintroduced at the end of each block. This ensured a sufficient number of trials for subsequent analysis and represented 16.9% of the trials in this experiment.

All participants underwent a short visual practice block (5 trials, 1 trial per target location) before starting the experiment to become familiar with the apparatus and procedure. The experiment ended with a recording of the 3D spatial position of the spherical targets using the same procedure as previously described. In total, the experiment lasted about 45 min.

#### Kinematic analyses

Due to signal loss, kinematic analyses were performed on the trajectories of 16 participants’ index finger. For each pointing movement, we extracted and analyzed off-line several kinematic parameters with a custom-made MATLAB program. We analyzed the latencies and amplitudes of the acceleration, velocity and deceleration peaks for the index finger’s tangential profile, namely from the combination of all three X, Y, Z axes. In order to assess the temporal dynamic of pointing movements toward the +45° and −45° targets, we additionally measured the latencies of the index finger’s acceleration, velocity and deceleration peaks on the horizontal (X) and vertical (Z) axes separately. This was done for the six conditions of interest, namely the +45° and −45° visual conditions, and the +45° and −45° congruent and incongruent verbal conditions.

To control for the appropriateness of our approach, we compared the kinematic values obtained for displacement on the horizontal axis (X) and those on the vertical axis (Z) with the kinematic values measured for the tangential displacement, for movements performed to the right, bottom and up targets in the visual block. As expected, data showed a major contribution of the X axis to the tangential profile (T) for pointing movements directed to the right target. Conversely, the Z-axis mainly contributed to the tangential profile of movements toward the targets located upward and downward (see Table 1).

**TABLE 1:** Latencies and amplitudes of the velocity peaks for tangential (T), vertical (Z) and horizontal (X) displacement of the participants’ index finger when pointing to visually cued targets located on the right, upwards and downwards.

Target location	Vel. Lat. T (ms)	Vel. Amp. T (mm/s)	Vel. Lat. Z (ms)	Vel. Amp. Z (mm/s)	Vel. Lat. X (ms)	Vel. Amp. X (mm/s)
Right	242	554	209	99	246	**536**
Up	221	537	222	**518**	253	74
Bottom	233	607	232	**591**	254	80

Vel. Lat., velocity peak latency; Vel. Amp., velocity peak amplitude. Values in bold indicate major contributions of the Z and X axes to the tangential profile for upward/downward targets and right targets respectively.

#### Statistics

We performed two types of statistical analyses to test our hypothesis of the primacy of verticality in movements. The first was based on the tangential profile of the pointing movements and aimed at determining the effect of Gravity (targets located at −45° vs. +45°) for the visual block and for the verbal block, the main effects of Gravity, Congruency (congruent vs. incongruent instructions) and their interaction in a 2 × 2 full factorial design. The visual and verbal blocks were analyzed separately but the analyses were similar. We first performed repeated measures permutation tests (Basso and Finos, 2012; Finos and Basso, 2014), with 5,000 random samplings, on each of the six kinematic parameters, namely the latency and amplitude of the acceleration, velocity, and deceleration peaks. The analysis of all those parameters is crucial to understand the unfolding movement and identify the motor program. To account for the multiplicity of these six univariate tests and control for the probability of false positives, we then conducted a non-parametric Fischer combination of these tests (Pesarin, 2001) to assess the effects of Gravity (in the visual and verbal blocks) and of Congruency (in the verbal block only) in a multivariate perspective. This test corresponds to a non-parametric MANOVA with repeated measures.

The second analysis aimed at examining for each of our six conditions of interest (i.e., −45° visual, +45° visual, −45° congruent, +45° congruent, −45° incongruent, and +45° incongruent), the temporal dynamic between the vertical (Z-axis) and horizontal (X-axis) components of the pointing movement and their relation to the tangential profile (T). For each condition, we calculated the difference (delta) between the latencies of the acceleration, velocity and deceleration peaks extracted from the X and from the tangential profiles (i.e., delta XT). We computed the same calculations for the Z profile with respect to the tangential one (i.e., delta ZT). We also directly compared the horizontal X and vertical Z components (i.e., delta XZ). This was done for the visual and verbal blocks separately. We then conducted the same univariate and multivariate analyses as described above for the three peak latencies, using the deltas as response variables.

In the following section, we first report the results of the tangential profile analysis (calculated on means of the latency and/or amplitude peaks) and then the results of the temporal dynamic analysis (calculated on deltas between the three axes). Statistical results for the Fischer combination tests are provided in the text whereas results of univariate analyses for each kinematic parameter are reported in the Figures’ legends.

### Results

#### Tangential profile

##### Gravity affected movement tangential profile, Congruency did not

**FIGURE 2:**
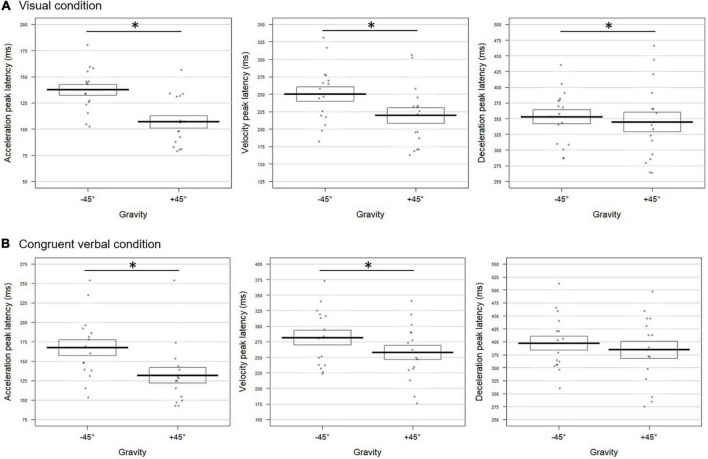
Gravity effect on the movement tangential profile for **(A)** visual and **(B)** congruent verbal instructions in Experiment 1. The bold lines represent peak latencies (in milliseconds) of the acceleration (left panel), velocity (middle panel), and deceleration (right panel) peaks averaged over all participants for movements toward –45° and +45° targets. Standard errors are illustrated by rectangles. Each dot represents the mean peak latency for one participant in the corresponding condition. Asterisk indicates a significant difference between conditions. **(A)** Upon visual instructions, acceleration, velocity, and deceleration peaked earlier for movements toward +45° than –45° targets (*t* = –7.72; *p* = 0.0004; *t* = –5.51; *p* = 0.0004; *t* = –2.42; *p* = 0.030, respectively). **(B)** For congruent verbal instructions (“en haut à droite”/*up right* and “en bas à droite”/*bottom right*), earlier acceleration and velocity peaks were found in the +45° than in the –45° condition (*t* = –6.65; *p* = 0.0004 and *t* = –4.03; *p* = 0.0028, respectively).

Pointing to a target located at +45° differed from pointing to a target at −45° both for visual and congruent verbal instructions. As can be seen from Figure 2A, movements performed toward +45° upon visual instructions were characterized by an acceleration peaking earlier (mean ± SD = 107 ± 23 ms) and of higher amplitude (4,742 ± 1,624 mm/s^2^, *t* = 2.95; *p* = 0.0068) than movements toward −45° (latency: 137 ± 21 ms; amplitude: 4,096 ± 1,265 mm/s^2^). Earlier velocity and deceleration peaks were also found for the +45° compared to the −45° condition (velocity: 219 ± 45 vs. 250 ± 41 ms, respectively; deceleration: 344 ± 62 vs. 353 ± 44 ms, respectively). The combined test performed on the *p*-values extracted for the six kinematic parameters revealed a highly significant effect of Gravity for the visual block (Fisher combination: *K* = 26.38; *p* = 0.001).

Albeit slightly less evident, similar effects of Gravity were observed in the verbal block, reaching significance on the time to acceleration and velocity peaks (Figure 2B). The two peaks occurred earlier in the +45° verbal condition with respect to the −45° one (acceleration: 127 ± 28 vs. 172 ± 61 ms; velocity: 254 ± 37 vs. 291 ± 55 ms for +45° and −45°, respectively). The combined effect of Gravity was significant (Fisher combination: *K* = 19.81; *p* = 0.012).

The factor Congruency did not significantly affect movement unfolding (for univariate tests, all *p*-values > 0.11; Fisher combination: *K* = 6.39; *p* = 0.36).

To sum up, in the visual and verbal blocks alike, fighting against gravity to reach the +45° target yielded to anticipated peaks of possibly higher amplitude with respect to movements at −45°.

#### Temporal dynamic

##### Visual and verbal congruent conditions: Vertical parameters occurred before horizontal ones for +45° targets

###### −45° visual

**FIGURE 3:**
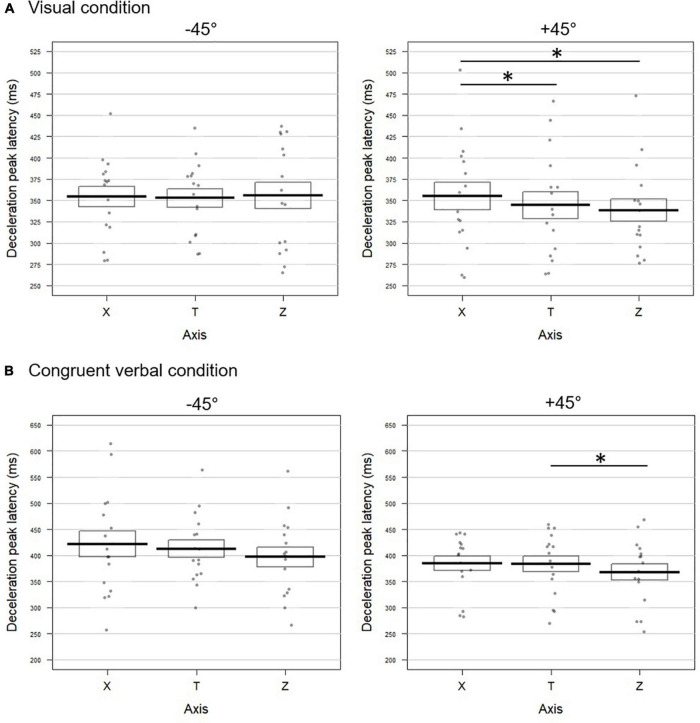
Temporal dynamic of the movements toward –45° (left panel) and +45° (right panel) targets upon **(A)** visual and **(B)** congruent verbal instructions in Experiment 1. Latencies (in milliseconds) of the deceleration peak along the horizontal (X), tangential (T), and vertical (Z) axes are reported. The bold lines represent peak latencies averaged across participants for each axis, rectangles illustrate the standard errors. Each dot stands for the mean peak latency for one participant in the corresponding condition. Significant difference between conditions are represented by *. **(A)** When pointing toward +45° visually cued targets, the deceleration peak occurred later on X than on T (*t* = 2.35; *p* = 0.032) and Z (*t* = –2.91; *p* = 0.016). **(B)** For movements toward +45° targets following verbally congruent instructions, earlier deceleration was found on Z than on T (*t* = –2.66; *p* = 0.018) and the effect approached significance for the comparison between Z and X (*t* = –2.01; *p* = 0.06).

The horizontal profile X was aligned with the tangential one, with no significant difference between the three kinematic parameters (all *p*-values > 0.4; Fisher combination: *K* = 1.27; *p* = 0.87; Figure 3A left panel for deceleration, and Supplementary Figure 1A for acceleration and velocity). The vertical acceleration profile showed a later peak both with respect to the tangential (delta TZ = 18.29 ms) and the horizontal components (delta XZ = 17.25 ms). Velocity and deceleration on Z did not differ neither from the tangential nor from the horizontal profiles (all *p*-values > 0.2), resulting in non-significant Fisher combinations (T vs. Z: *K* = 5.8; *p* = 0.1; Z vs. X: *K* = 6.06; *p* = 0.09).

###### +45° visual

The latencies of the acceleration, velocity and deceleration peaks extracted from the horizontal profile (X) occurred significantly later than those calculated on the tangential profile T (delta TX for acceleration: 12.90 ms; velocity: 19.70 ms; deceleration: 10.98 ms; Figure 3A right panel and Supplementary Figure 1A). The combined effect on these three kinematic parameters for the comparison between X and T was significant (Fisher combination: *K* = 12.95; *p* = 0.0052). None of the peak latencies extracted from the vertical axis (Z) significantly differed from the tangential profile (all *p*-values < 0.2; Fisher combination: *K* = 2.86; *p* = 0.41). Accordingly, the velocity and deceleration peaks occurred earlier on the vertical axis with respect to the horizontal one (delta XZ for velocity: 22.83 ms; deceleration: 16.62 ms; see Figure 3A and Supplementary Figure 1A). The combined effect for the XZ comparison also reached significance (Fisher combination: *K* = 11.81, *p* = 0.01).

###### −45° congruent verbal

Confirming the pattern obtained in the visual block, the latencies of the acceleration, velocity, and deceleration peaks as observed on the X profile did not significantly differ from those calculated on the tangential one (all *p*-values > 0.2 for univariate tests; Fisher combination: *K* = 2.22; *p* = 0.64; Figure 3B left panel for deceleration and Supplementary Figure 1B for the two other parameters). The same pattern was observed for the peak latencies on Z (all *p*-values > 0.3; Fisher combination: *K* = 1.40; *p* = 0.76). Similarly, we found no difference between the X and Z latencies on any of the three kinematic parameters (all *p*-values > 0.09; Fisher combination: *K* = 3.50; *p* = 0.31).

###### +45° congruent verbal

The acceleration peak extracted from the horizontal profile occurred significantly later than the tangential one (delta TX = 17.21 ms; Supplementary Figure 1B; Fisher combination for T vs. X: *K* = 5.56; *p* = 0.1). On the contrary, as shown in Figure 3B (right panel) and Supplementary Figure 1B, the velocity and deceleration peaks on the vertical axis occurred earlier (delta TZ = 11.30 and 12.78 ms, respectively) than those of the tangential profile, resulting in a significant combined effect over the three peak latencies (Fisher combination: *K* = 10.30; *p* = 0.012). Coherent with this pattern, the kinematic parameters on the vertical axis occurred earlier than those measured on the horizontal one (delta XZ for acceleration: 20.84 ms; velocity: 19.80 ms; and deceleration: 14.67 ms), with a significant combined effect (Fisher combination: *K* = 11.38; *p* = 0.012; Figure 3B and Supplementary Figure 1B).

##### Incongruent verbal conditions: Vertical and horizontal parameters aligned for +45° targets

###### −45° incongruent verbal

We did not observe any misalignment from the tangential profile neither on the horizontal nor on the vertical axes (for univariate analyses, all *p*-values > 0.1). However, the deceleration peak occurred significantly earlier on the vertical than on the horizontal axis (delta XZ = 20.55 ms). None of the Fisher combinations resulted significant (T vs. X: *K* = 2.70; *p* = 0.51; T vs. Z: *K* = 2.59; *p* = 0.43; X vs. Z: *K* = 5.1; *p* = 0.14).

###### +45° incongruent verbal

Similar to −45°, none of the profiles significantly differed from each other (all *p*-values > 0.08; (Fisher combinations: T vs. X: *K* = 4.6; *p* = 0.19; T vs. Z: *K* = 4.91; *p* = 0.16; X vs. Z: *K* = 6.4; *p* = 0.1). In other words, hearing incongruent instructions mildly perturbed the temporal dynamic of the movement, resulting in an alignment of the horizontal and vertical kinematic parameters with the tangential profile.

Overall, when gravity was not an issue, namely when participants pointed to the −45° target, irrespective of the condition (visual, congruent verbal, or incongruent verbal), the horizontal and vertical parameters remained aligned with the tangential and did not differ from each other. Most importantly, under visual and canonical verbal instructions, when the movement was directed against gravity, toward the +45° target, the horizontal profile exhibited delayed peaks with respect to the tangential profile. On the contrary, kinematic parameters occurred earlier on the vertical axis with respect to the tangential and/or the horizontal profile. Non-canonical verbal instructions disturbed the organization of +45° movements, resulting in an alignment between the horizontal and vertical axes both with the tangential and among them.

In this first experiment, verbal instructions that were incongruent regarding Path expression in French (“à droite en haut”) did not affect the general tangential profile of the movement, however they subtly altered the temporal dynamic linking the horizontal and vertical axes. Nevertheless, the effect was not sufficient to entirely reverse the kinematic pattern with respect to the congruent verbal condition. Kinematic parameters on the horizontal axis were indeed not found to occur earlier than those on the vertical or the tangential profiles. To further assess whether movement temporal dynamic is flexible and shows plasticity for language or whether it is immune to it, we conducted a second experiment which was comparable to the first one in all respects except in the timing of the movement with respect to verbal instructions. Whereas in Experiment 1 the go signal for the pointing movement was a visual cue delivered after verbal instruction offset, participants were not required to wait for the instructions’ offset to start their movement in Experiment 2.

## Experiment 2

### Materials and methods

#### Participants

A new group of 19 French native healthy volunteers [12 females, 24 ± 2.7 years old, age range (21–30 years old)] was recruited to participate in Experiment 2. None of them had participated in the first experiment. The participants fulfilled the same inclusion criteria as defined in Experiment 1 and they were naive to the purpose of the study. All participants signed an informed consent prior to the experiment, which conformed to the declaration of Helsinki and was approved by a national ethics committee (Comité de Protection des Personnes Sud-Est II). The participants received monetary compensation for their participation.

#### Apparatus and stimuli

The apparatus and stimuli were identical to those used in Experiment 1.

#### Procedure

The procedure of the visual block was exactly the same as in the first experiment. Only the procedure of the verbal block was slightly different regarding the go signal for the pointing movement. After the gauge ended and a variable delay of 700 to 1,000 ms, the auditory verbal instruction was delivered to the participants. This was the go signal, namely they were required to point to the designated target as fast and accurately as possible. This procedure therefore differed from that of Experiment 1 in that participants did not have to wait until the very end of the verbal instruction to perform their movements. The remaining procedure was comparable to that of Experiment 1. The % of trials reintroduced at the end of the visual and verbal blocks due to anticipated movements, unvalidated targets or targets unreached within the allocated time was 10.9%.

#### Kinematic and statistical analyses

The kinematic and statistical analyses were similar in all respects to those in Experiment 1.

### Results

#### Tangential profile

##### Gravity affected movement tangential profile

**FIGURE 4:**
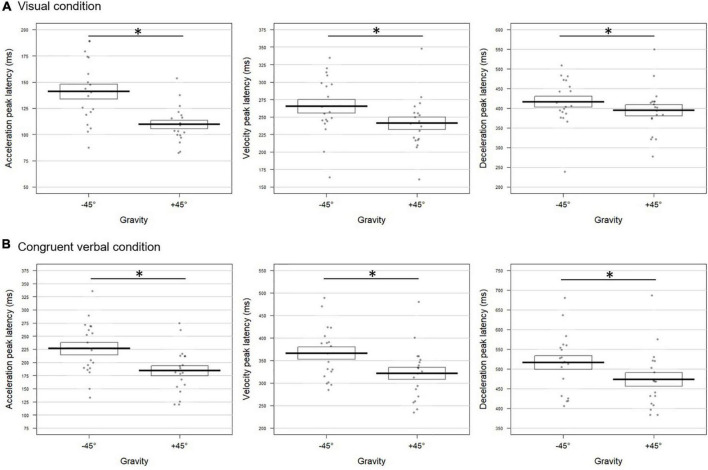
Gravity effect on the movement tangential profile for **(A)** visual and **(B)** verbally congruent instructions in Experiment 2. Latencies of the acceleration (left panel), velocity (middle panel), and deceleration (right panel) peaks are reported for movements toward –45° and +45° targets (see Figures 2, 3 for conventions; * indicates a significant difference between conditions). **(A)** For visually cued targets, acceleration, velocity, and deceleration peaked earlier in the +45° than in the –45° condition (*t* = –10.07; *p* = 0.0004; *t* = –6.56; *p* = 0.0004; and *t* = –5.16; *p* = 0.0004, respectively). **(B)** Upon congruent verbal instructions, the three kinematics parameters also occurred earlier when participants pointed toward +45° compared to –45° targets (acceleration: *t* = –4.79; *p* = 0.0012; velocity: *t* = –5.17; *p* = 0.0008; deceleration: *t* = –3.59; *p* = 0.0004).

When considering the effect of Gravity, we observed a similar pattern to that of Experiment 1 (Figure 4A for the visual block and Figure 4B for the verbal block). In both the visual and verbal blocks, pointing to targets located at +45° led to significantly earlier acceleration compared to movements toward −45° targets (mean ± SD for the visual block: 110 ± 17 vs. 141 ± 30 ms; verbal block: 151 ± 34 vs. 220 ± 66 ms for +45° and −45°, respectively). Similarly, velocity and deceleration peaks occurred earlier for +45° targets than for −45° ones (visual block velocity: 241 ± 38 vs. 265 ± 43 ms and deceleration: 395 ± 60 vs. 416 ± 61 ms for +45° and −45°, respectively; verbal block velocity: 293 ± 52 vs. 362 ± 71 ms and deceleration: 452 ± 86 vs. 509 ± 88 ms, respectively). In addition, for the visual and verbal blocks alike, the acceleration peak was of higher amplitude in the +45° condition than in the −45° condition (visual: 4,118 ± 1,157 vs. 3,518 ± 1,220 mm/s^2^, *t* = 4.67; *p* = 0.0004 and verbal: 3,700 ± 859 vs. 3,059 ± 932 mm/s^2^, *t* = 5.65; *p* = 0.0004). The velocity peak amplitude was also higher for +45° than for −45° movements but only in the verbal block (503 ± 75 vs. 474 ± 81 mm/s, respectively, *t* = 2.54; *p* = 0.01), the effect being marginally significant in the visual block (*t* = 2.01; *p* = 0.055). Similarly, the effect on the deceleration peak amplitude only approached significance in both blocks (visual: *t* = −2.01; verbal: *t* = −1.97; both *p* < 0.063). The combined effect of Gravity for the six kinematic parameters was significant for each of the two blocks (visual block: Fisher combination: *K* = 36.98, *p* = 0.0008; verbal block: Fisher combination: *K* = 36.21; *p* = 0.0008).

##### Non-canonical verbal instructions delayed movement tangential profile

**FIGURE 5:**
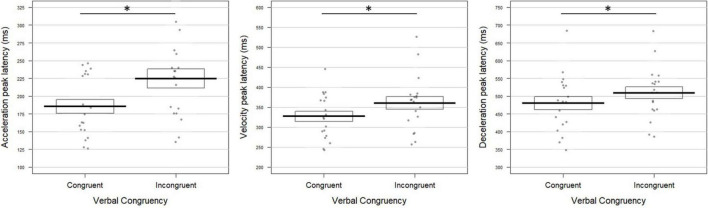
Verbal congruency effect on the movement tangential profile in Experiment 2. Latencies (in milliseconds) of the acceleration **(left panel)**, velocity **(middle panel)**, and deceleration peaks **(right panel)** were longer when pointing toward both upward (+45°) and downward (–45°) targets upon incongruent (“à droite en haut”/*right up* and “à droite en bas”/*right bottom*) with respect to congruent (“en haut à droite”/*up right* and “en bas à droite”/*bottom right*) verbal instructions (acceleration: *t* = –3.40; *p* = 0.003; velocity: *t* = –3.05; *p* = 0.005; deceleration: *t* = –2.21; *p* = 0.031). The conventions are the same as in Figure 2. The symbol * indicates a significant difference between conditions.

As illustrated in Figure 5, congruency significantly affected all the parameters’ latency, resulting in delayed peaks for non-canonical verbal instructions (acceleration: 225 ± 62 ms; velocity: 361 ± 72 ms; and deceleration: 510 ± 79 ms) with respect to canonical ones (acceleration: 186 ± 50 ms; velocity: 327 ± 61 ms; and deceleration: 480 ± 87 ms). The combined effect was also significant (Fisher combination: *K* = 19.72; *p* = 0.0052). The effects of Congruency were furthermore increased in the +45° condition with respect to −45° as underlined by a significant interaction between the two factors on the acceleration and velocity peaks (Interaction Gravity × Congruency for acceleration latency: *t* = −2.61; *p* = 0.009; velocity latency: *t* = −2.49; *p* = 0.016; deceleration amplitude: *t* = −2.06; *p* = 0.048; combined effect: Fisher combination: *K* = 15.71; *p* = 0.020).

As in Experiment 1, moving toward the +45° target was effortful, which led to an anticipation of the kinematic parameters possibly accompanied by an increase in their amplitude. Most interestingly, incongruent, non-canonical verbal instructions delayed movement kinematic parameters: the acceleration, velocity and deceleration peaks were reached later in the incongruent condition. This effect was however stronger for pointing movements performed against the gravity, that is to a target located at +45°.

#### Temporal dynamic

##### Visual and verbal congruent conditions: Vertical parameters occurred before horizontal ones when fighting against gravity

###### −45° visual

**FIGURE 6:**
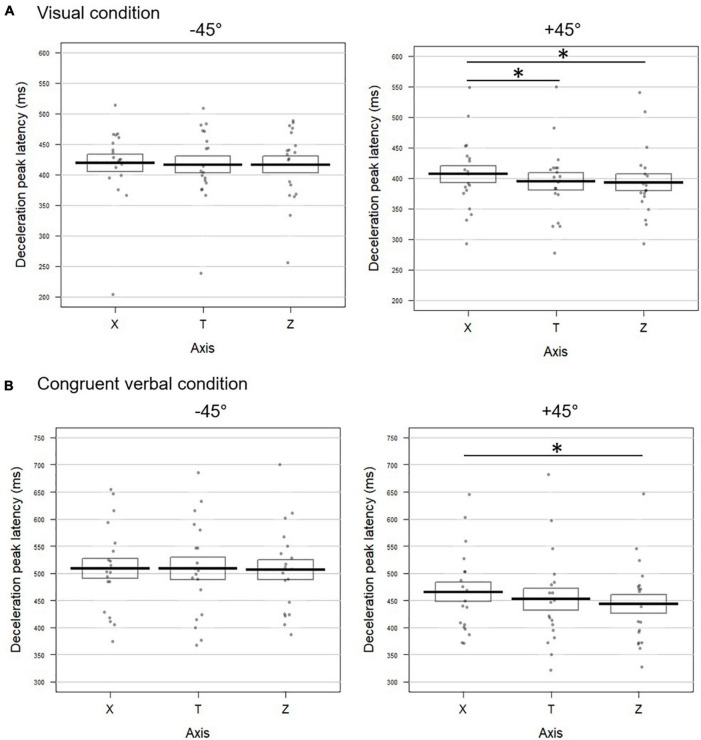
Temporal dynamic of the movements toward –45° (left panel) and +45° (right panel) targets upon **(A)** visual and **(B)** congruent verbal instructions in Experiment 2. Latencies (in milliseconds) of the deceleration peak along the horizontal (X), tangential (T), and vertical (Z) axes are reported (see Figure 3 for conventions; * indicates a significant difference between conditions). **(A)** In the visual block, pointing toward +45° targets resulted in later deceleration on X than on T (*t* = 2.29; *p* = 0.038) and on Z (*t* = –2.68; *p* = 0.016). **(B)** For +45° targets upon verbally congruent instructions, the deceleration peak occurred earlier on Z than on X (*t* = –4.18; *p* = 0.0008).

When participants pointed to targets at −45° upon visual instructions, the horizontal profile was aligned with the tangential one with no significant difference in the timing of their kinematic parameters (all *p*-values > 0.68 for univariate analyses; T vs. X Fisher combination: *K* = 0.78; *p* = 0.95; see Figure 6A left panel for deceleration and Supplementary Figure 2A for acceleration and velocity). The vertical acceleration profile showed a later peak both with respect to the tangential (delta ZT = 21.57 ms) and the horizontal profiles (delta XZ = 23.52 ms). Velocity and deceleration on Z did not significantly differ neither from the tangential profile nor from the horizontal axis (all *p*-values > 0.4). Despite the effects were limited to one out of the three kinematic parameters, both Fisher combination tests reached significance (Z vs. T: *K* = 8.70; *p* = 0.0096; Z vs. X: *K* = 7.86; *p* = 0.016).

###### +45° visual

As shown in Figure 6A (right panel) and Supplementary Figure 2A, for movements toward +45° visual targets, the acceleration, velocity, and deceleration peaks extracted from the horizontal profile (X) occurred significantly later than those calculated on the tangential profile (delta XT for acceleration: 13 ms; velocity: 11.33 ms; and deceleration: 11.31 ms). The combined effect on the three peak latencies was significant (Fisher combination: *K* = 15.66; *p* = 0.0004). None of the latencies extracted from the vertical axis (Z) differed from the tangential profile (all *p*-values > 0.16; Fisher combination: *K* = 2.66; *p* = 0.49). The direct comparison between X and Z revealed that the three kinematic parameters peaked significantly earlier for the vertical profile with respect to the horizontal one (delta XZ for acceleration: 13.76 ms; velocity: 14.85 ms; deceleration: 14.21 ms; and Fisher combination: *K* = 13.58; *p* = 0.002).

###### −45° congruent verbal

The latencies of the acceleration, velocity and deceleration peaks on the X profile did not significantly differ from those calculated on the tangential profile (all *p*-values > 0.2 for univariate tests; Fisher combination: *K* = 1.75; *p* = 0.72; Figure 6B left panel for deceleration and Supplementary Figure 2B for the other parameters). The same pattern was observed for the Z peak latencies (all *p*-values > 0.5; Fisher combination: *K* = 1.23; *p* = 0.85). No difference was observed between the latencies of the three peaks when comparing the horizontal and vertical components (all *p*-values > 0.4; Fisher combination: *K* = 1.47; *p* = 0.77).

###### +45° congruent verbal

Two out of the three kinematic parameters on the horizontal axis were significantly delayed with respect to the tangential profile (delta XT for acceleration: 15.15 ms; velocity: 19.17 ms), yielding a significant combined effect (Fisher combination: *K* = 15.94; *p* = 0.0004; Figure 6B right panel and Supplementary Figure 2B). Conversely, on the vertical axis, the velocity peak occurred 10 ms earlier than on the tangential profile. The combined test for the comparison between Z and T also revealed a significant effect (Fisher combination: *K* = 8.78; *p* = 0.011). Finally, the three kinematic parameters on the vertical profile occurred significantly earlier than those recorded on the horizontal profile (delta XZ for acceleration: 20.87 ms; velocity: 29.12 ms; deceleration: 21.95 ms; Fisher combination: *K* = 20.69; *p* = 0.0004).

##### Incongruent verbal conditions: Incongruency altered the temporal dynamic of the movement

###### −45° incongruent verbal

**FIGURE 7:**
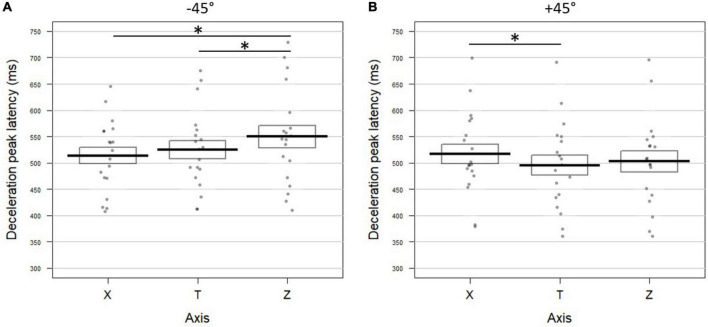
Effect of verbal incongruency on the temporal dynamic of movements toward **(A)** –45° and **(B)** +45° targets in Experiment 2. Latencies (in milliseconds) of the deceleration peak extracted from the horizontal (X), tangential (T), and vertical (Z) axes when hearing an incongruent verbal instruction (**A:** “à droite en bas”/*right bottom*; **B:** right panel: “à droite en haut”/*right up*; see Figure 6 for conventions; * indicates a significant difference between conditions). **(A)** When pointing downward toward –45° targets, deceleration peaked later on Z than on T (*t* = 3.13; *p* = 0.003) and on X (*t* = 3.00; *p* = 0.002). **(B)** For movements upward to +45° targets, the deceleration peak occurred later on X than on T (*t* = 2.42; *p* = 0.024), yet the Fischer combination test on all parameters was not significant (T vs. X: *K* = 5.62; *p* = 0.1).

The acceleration peak on the horizontal axis occurred 10 ms earlier than the one on the tangential profile; yet the Fisher combination did not reach significance (*K* = 5.14; *p* = 0.12; Figure 7A for deceleration and Supplementary Figure 3 for the other kinematic parameters). On the vertical axis, the acceleration, velocity and deceleration peaks occurred later than the tangential ones (delta ZT for acceleration: 30.83 ms; velocity: 26.57 ms; and deceleration: 25.77 ms). This resulted in a significant combined effect (Fisher combination: *K* = 19.31; *p* = 0.0004). In line with this pattern and in contrast to all the previously reported results, the three kinematic parameters on the vertical profile were delayed with respect to those recorded on the horizontal axis (delta XZ for acceleration: 56.37 ms; velocity: 28.31 ms; deceleration: 35.76 ms; Fisher combination: *K* = 18.05; *p* = 0.0004).

###### +45° incongruent verbal

In the incongruent +45° condition, we found only two differences between the kinematic parameters of the three profiles. With respect to the tangential profile, the deceleration peak was delayed by 23.65 ms for the horizontal axis (Figure 7B) and by 7.67 ms for the acceleration peak on the vertical axis (Supplementary Figure 3 right panel). The horizontal temporal dynamic did not differ from the vertical one (all *p*-values > 0.07 for univariate tests). None of the combined tests reached significance (T vs. X: *K* = 5.62; *p* = 0.1; T vs. Z: *K* = 5.7; *p* = 0.1; X vs. Z: *K* = 5.11; *p* = 0.14).

When scrutinizing the temporal dynamic along the horizontal and vertical axes, the patterns observed for visual and canonical verbal instructions accurately reproduced the results in the first experiment. When participants pointed downward to the −45° target, the misalignment with the tangential profile was absent or anecdotal. In striking contrast, moving upward to the +45° target induced a delay on the horizontal profile, while the vertical axis remained anchored onto the temporal dynamic exhibited by the tangential profile. When an incongruent verbal instruction was delivered, movements fighting against gravity (+45°) were mildly perturbed, mostly leading to an alignment between the horizontal and vertical parameters with the tangential component and among them. This pattern replicated the results from Experiment 1. Most importantly, movements directed downward (−45° target) following a non-canonical verbal instruction were deeply affected in their temporal dynamic: the kinematics of the vertical profile was markedly delayed with respect to that of both the horizontal and tangential profiles.

## Discussion

In light of the embodiment of language in action, the present study sought to investigate whether the primacy of verticality in Path expression across various languages is also reflected in the organization of biological movements. To this aim, we examined the kinematics and temporal dynamic of pointing movements toward spatial locations that feature horizontal and vertical components alike (up right +45° and bottom right −45°). As a second aim, we tackled whether and how language can affect this organization by assessing the influence of non-canonical verbal instructions that do not prioritize verticality (“à droite en haut”/*right up*) on movement kinematics.

Results from two experiments in two groups of healthy adults first showed a massive effect of gravity on movement kinematics. Upon both visual and canonical verbal instructions, kinematic parameters of movements directed to +45° targets were significantly anticipated with respect to movements at −45°, as reflected by earlier acceleration, velocity and/or deceleration peaks. The amplitude of the acceleration and velocity peaks was furthermore higher in the +45° condition depending on the experiment and block (visual or verbal). This pattern of results agrees with previous findings of kinematic asymmetries for upward and downward movements of the upper limb (Papaxanthis et al., 1998, 2003; Le Seac’h and McIntyre, 2007; Berret et al., 2008; Crevecoeur et al., 2009; Yamamoto et al., 2019; Poirier et al., 2020). Gentili et al. (2007) showed shorter duration and higher amplitude of acceleration for pointing movements toward targets located upward vs. downward, whereas similar profiles were observed for left-right movements. Gaveau and Papaxanthis (2011) reported similar duration for up and down movements but different temporal dynamic of acceleration profiles, the latter being performed faster. Interestingly, such kinematic differences attenuate in microgravitational environments (Papaxanthis et al., 2005; Gaveau et al., 2016). Anticipated acceleration and/or velocity peaks possibly combined with higher peak amplitudes are indicative of more effortful movements. Along this line, previous work demonstrated acceleration peak of shorter latency when participants reached and grasped heavy objects as compared to lighter ones (Roy et al., 2013; Martel et al., 2020). Our findings thus confirm that performing movements upward, against gravitational forces that constantly pull the body downward, is costly and implies different motor planning strategies with respect to downward movements. Pointing to upward targets indeed requires to integrate gravitational constraints so as to optimize the motor commands while saving muscle effort (Gentili et al., 2007; Gaveau et al., 2016, 2021). Interestingly, typological analyses of motion events in different languages reveal that the effort expended by the moving Figure to overcome gravity is typically coded vertically (Łozińska, 2021). The saliency of verticality in language is also attested by the frequent usage of conceptual metaphors that express abstract ideas along the vertical dimension (Lakoff and Johnson, 1999, 2003; Gallese and Lakoff, 2005; Cian, 2017). In this regard, pitch is metaphorically conceptualized in the vertical space in most Western European languages and some non-Indo-European languages, with high pitch associated with upward movements and upper space, and low pitch associated with downward movements and lower space (Eitan and Timmers, 2010; Clark et al., 2013; Fernandez-Prieto et al., 2017; Holler et al., 2022; see Walker et al., 2010 and Dolscheid et al., 2014 for evidence in prelinguistic infants, and Shayan et al., 2011 and Dolscheid et al., 2013 for thickness/pitch association in languages such as Farsi and Turkish). In a speeded pitch discrimination task, Rusconi et al. (2006) revealed faster and more accurate responses to decide that tones where higher or lower in frequency than a reference tone when participants pressed an upper or lower key, respectively. Morett et al. (2022) furthermore reported that when English speakers learned pitch contours of Mandarin lexical tones with pitch gestures and dot motion congruent with the conceptual metaphor of pitch, performance was better than when learning involved incongruent pitch gestures or motionless dots (see also Morett and Chang, 2015). Pitch-varying stimuli have also been shown to influence visual motion perception (Maeda et al., 2004): ambiguous motion stimuli were perceived as moving upward when presented together with ascending pitch pure tones, but downward when combined with descending pitch sounds (see also Connell et al., 2013 for effects of observing upward or downward manual gestures). Metaphors anchoring concepts along the vertical dimension are also frequently used for emotional valence: words associated with positive experiences are quasi-universally mapped onto the upper space whereas the lower space is more dedicated to negative experiences (Meier and Robinson, 2004; Kövecses, 2008; Sasaki et al., 2016; Cian, 2017; but see Wnuk and Ito, 2021 for metaphors mapping up to undesirable and down to desirable events in Mlabri, an Austroasiatic language of Thailand and Laos). Generating words associated with disappointment has for instance been shown to decrease the posture height of participants more than generating words related to pride (Oosterwijk et al., 2009). Similarly, power has been shown to be represented along a vertical plane with powerfulness at the top (Jiang and Henley, 2012). Participants were faster to judge if a social group was more powerful (e.g., “masters” vs. “servants”) when the powerful group was located in the upper part of the screen (Schubert, 2005). In line with the grounding of language in action hypothesis and since effort is socially valued, the association “positive is up” could be rooted in the cost of upward movements.

Crucially for our purpose, analysis of the temporal dynamic of arm pointing movements highlighted a remarkably consistent pattern across our two experiments and across the visual and verbal blocks. When participants reached targets located at +45° from their starting position, irrespective of whether instructions were visual or canonical, kinematic parameters extracted from the horizontal X-axis were systematically delayed from those on the tangential profile. On the contrary, parameters on the vertical Z-axis either aligned with or occurred earlier than those measured on the tangential component. Most notably, direct comparison between the temporal dynamic of the vertical and horizontal axes for the two conditions under scrutiny (+45° visual and +45° congruent verbal) revealed that displacement on the vertical plane was always anticipated with respect to displacement on the horizontal plane. This finding strikingly parallels observations from semantic typology that languages from different families express elements encoding vertical Path before those elements encoding horizontal displacement (Imbert, 2013). Studies in various languages also revealed asymmetries between verbs that express motion along the vertical and the horizontal axes. In Polish and Russian for instance, verbs for vertical spatial relations are more frequently coded in verbal descriptions than verbs for horizontal motion. This may stem from the perceptual difference between horizontal and vertical biological movements, the latter not being canonical among most animate entities, which may in turn affect linguistic structures (Łozińska, 2018, 2021). The strategies for expressing vertical relations also diverge from those used for horizontal relations, both in verb- and satellite-framed languages. Verb-framed languages (e.g., Spanish and French) typically encode Path in the main verb, whereas satellite-framed languages (e.g., English and Polish) do it in grammatical elements (i.e., preverbs or particles) with Manner of motion being expressed in the main verb (Talmy, 2000). However, studies have revealed that when it comes to vertical motion, the pattern diverges from what would be expected from the languages’ typology. Speakers indeed tend to encode vertical motion in Manner verbs in verb-framed languages and in Path verbs in satellite-framed languages (Naigles et al., 1998; Łozińska and Pietrewicz, 2018).

Our kinematic results provide novel evidence for shared processes between biological movements and language by revealing that both functions organize trajectories following the same rule, namely by giving priority to verticality. As already outlined, this primacy of the vertical axis may pertain to the fundamental importance of gravity in our everyday life (White et al., 2020). Senot et al. (2005) for instance reported that participants adjusted their motor response to catch a ball depending on motion direction with respect to the vertical plane: movement started earlier when the ball came from above rather than from below. Gravity also strongly affects the perception of biological motion so that inverting the stimuli upside down alters accurate motion recognition (Troje and Westhoff, 2006; Wang et al., 2022). The present study suggests that verticality is so deeply anchored in our brain that it shapes action and language similarly. In agreement with previous typological reports on the verbal description of motion events, we show that when performing upward pointing movements that imply both horizontal and vertical vectors, priority is given to displacement along the vertical axis. It is noteworthy that this pattern was only observed for movements fighting against gravity, the temporal dynamic being markedly different for −45° movements. In this condition, parameters extracted from the horizontal and vertical profiles were indeed aligned with those from the tangential one. These results are reminiscent of previous studies showing differential effects for up and down movements. Crevecoeur et al. (2009) found stronger effects of hypergravity when participants moved upward than downward. In the field of psycholinguistics, Meteyard et al. (2007) reported contrasting results depending on the direction of motion: whereas listening to incongruent motion-related words increased response times to detect upward motion in visual stimuli, response times were decreased for downward motion.

To assess the potential imprint of language on action, we finally examined how non-canonical verbal instructions that do not obey the primacy of verticality affected movement kinematics. No effect was found on the tangential profile in Experiment 1 when the pointing movement was triggered after the offset of the incongruent verbal condition. However, in the second experiment, namely when participants could start their movement while the verbal instruction was still ongoing, our findings revealed a significant effect of verbal congruency on this same tangential profile: movements performed in response to incongruent instructions displayed delayed kinematic parameters. The differential effects on tangential profiles may result from the difference in the timing of lexico-semantic access between the two experiments: participants had fully accessed the verbal instruction at movement onset in Experiment 1 while lexico-semantic processing was still ongoing in Experiment 2, thus interfering with concurrent movement execution. This agrees with previous work showing that word and sentence processing can influence simultaneous motor responses (as observed on the tangential profile) and underlines the crucial importance of the relative timing between linguistic and motor tasks for the cross-talk between the two to occur (Gentilucci and Gangitano, 1998; Gentilucci et al., 2000; Borreggine and Kaschak, 2006; Boulenger et al., 2006, 2008; Zwaan and Taylor, 2006; Meteyard et al., 2007; Kaschak and Borreggine, 2008; Nazir et al., 2008). As an alternative, the influence of verbal instructions on the movement tangential profile might be restricted to a limited time-window. The potential effect of incongruency would therefore have been canceled out when participants had to wait for the offset of the verbal stimuli to start their movement. However, this interpretation is unlikely for two reasons. First, the incongruency effect on the global structure of the movement (i.e., tangential profile) in Experiment 2 was still observed on the deceleration peak (at a mean latency of ∼500 ms from movement onset), arguing against a short-lived phenomenon. Second, non-canonical verbal instructions affected, although in a different form, the movement temporal dynamic along the horizontal and vertical axes in both Experiments 1 and 2. Overall our findings therefore suggest that non-canonical verbal instructions may affect two levels of movement execution: while the tangential profile could resist non-canonical instructions provided they were not concurrent with movement execution, the fine-grained temporal dynamics linking the different axes revealed to be most sensible to verbal instructions.

Convincing evidence exists regarding congruency effects between words referring to verticality (e.g., climb) and either their position in space (e.g., upper space) or vertical movements of the body (e.g., upward movement; Zwaan and Yaxley, 2003; Brookshire et al., 2010; Casasanto and Dijkstra, 2010; Dudschig et al., 2015; Lachmair et al., 2016; Globig et al., 2019). These effects also hold for metaphorical meaning. Evaluation of the positive (e.g., hero) or negative (e.g., liar) valence of words is faster if words are displayed at the top or the bottom of the screen, respectively (Meier and Robinson, 2004). In the so-called action compatibility effect, Santana and de Vega (2011) furthermore showed faster responses to judge sentences such as “The pressure gas made the balloon rise” (literal) and “His talent for politics made him rise to victory” (metaphorical) when the direction of the motor response matched the direction in the motion verbs. Finally, in a study using electroencephalography (EEG), words referring to upper or lower spatial positions either literally (e.g., ceiling/ascend or floor/descend) or metaphorically (e.g., power/inspire, or defeat/poverty) elicited more positive evoked brain responses when accompanied by incongruent manual movements than by congruent ones (Bardolph and Coulson, 2014). For literal words, this effect occurred early after word onset (∼200–300 ms), suggesting stronger motor activity to access word meaning in the incompatible condition. This is in line with the time-course of action word lexico-semantic processing and of motor/language interference found in previous work (Pulvermüller et al., 1999; Hauk and Pulvermüller, 2004; Boulenger et al., 2006). Effects for metaphorical words occurred later, after initial access to meaning (from 500 to 700 ms after word onset), which the authors interpreted as reflecting additional cognitive cost to integrate words associated with incongruent vertical motion (see Brouwer et al., 2012).

The second major finding regarding our congruency effect pertains to the temporal dynamic of the movement. As a reminder, in both experiments, upon visual and canonical verbal cues, kinematic parameters from the vertical Z-axis always occurred before those from the horizontal X-axis. Remarkably, when participants heard a non-canonical verbal instruction, this pattern was no longer observed. For movements toward +45° targets (up right), parameters of the vertical and horizontal profiles became aligned with those of the tangential upon hearing of “à droite en haut” (*right up*). But the most striking effects were obtained when participants pointed to targets located bottom right, at −45°. In this condition, and only in this condition, hearing a verbal instruction that was not canonical with the primacy of verticality in motion description (“en bas à droite” *right bottom*) completely inverted the temporal dynamic of the movement. Acceleration, velocity and deceleration peaks along the vertical axis occurred later than those along the horizontal axis. In other words, when priority was no longer given to verticality in language, the vertical axis stepped aside in favor of the horizontal axis to follow the temporality of the verbal instruction. This finding first substantiates the flexibility of the motor system that has been reported in studies varying the effects of gravity (see White et al., 2020 for a review) as well as in the previously described studies examining interactions with language (Gentilucci and Gangitano, 1998; Gentilucci et al., 2000; Boulenger et al., 2006). Most notably, our data suggest that the motor system may be more permeable to incongruency in linguistic Path expression when movements are performed downward than upward. As previously stated, upward movements require more energy and effort to counteract gravitational forces for optimal motor performance (Gentili et al., 2007; Gaveau et al., 2016). The predominance of the vertical axis with respect to the horizontal one in the temporal dynamic of +45° movements is deeply grounded, as highlighted in our two experiments. Accordingly, a non-canonical verbal instruction can only affect this dynamic to a limited extent, not to the point of totally reversing it: differences between the three axes of the kinematic profile are erased. In other words, when hearing “à droite en haut” (*right up*), parameters along the vertical axis do no longer precede those along the horizontal axis but they are not sufficiently delayed so as to peak later. On the contrary, downward movements, going in the direction of gravity, require less effort and do not show the same temporal asymmetries as upward movements in the case of visual and canonical verbal cues (i.e., alignment of the vertical and horizontal axes with the tangential profile). This condition may therefore appear more permeable to changes in the verbal expression of Path: hearing “à droite en bas” (*right bottom*) leads to anticipated kinematic parameters along the horizontal profile with respect to the vertical one. To put it differently, only movements that do not fight against gravity allow for verticality to come after horizontality in case of non-canonical verbal instructions. Such findings raise the intriguing question of whether some of the world’s languages show flexibility in the order of horizontal and vertical morphemes when encoding a downward Path.

## Conclusion

In conclusion, the present study reaffirms the constraints gravity impose on biological movements, thus echoing the typological differences in vertical and horizontal motion descriptions. Most importantly, we provide first evidence to our knowledge for the primacy of vertical encoding in the execution of hand pointing movements, as reported in linguistic typology on Path expression. Finally, we demonstrate that following non-canonical verbal instructions prioritizing the horizontal axis, the movement temporal organization may reverse as long as fighting gravity is no longer the priority. Overall our findings shed new light on the embodiment of language by revealing that linguistic Path may reflect the organization of biological movements.

## Data availability statement

The raw data supporting the conclusions of this article will be made available by the authors, without undue reservation.

## Ethics statement

The studies involving human participants were reviewed and approved by the Comité de Protection des Personnes Sud Est II. The patients/participants provided their written informed consent to participate in this study. Written informed consent was obtained from the individual(s) for the publication of any potentially identifiable images or data included in this article.

## Author contributions

VB and AR conceptualized and designed the study, conducted the research, and wrote the original draft. CD, RS, and EK contributed to methodology. CD helped in data acquisition. LF performed the statistical analysis. All authors contributed to the article and approved the submitted version.

## Footnotes

1
https://www.crnl.fr/en/plateforme/neuro-i

2
https://www.oculus.com

## Supplementary material

Supplementary Figure 1 Temporal dynamic of the movements toward −45° and +45° targets upon **(A)** visual and **(B)** congruent verbal instructions, for the acceleration (left panel) and velocity (right panel) peaks in Experiment 1. Latencies (in milliseconds) of the peaks along the horizontal (X), tangential (T), and vertical (Z) axes are reported. The bold lines represent peak latencies averaged across participants for each axis, rectangles illustrate the standard errors. Each dot stands for the mean peak latency for one participant in the corresponding condition. Asterisk indicates a significant difference between the conditions. **(A)** Upon visual cues, when participants pointed toward −45° targets, acceleration peaked later on Z than on X (*t* = 3.10; *p* = 0.011) and T (*t* = 2.98; *p* = 0.014). For +45° targets, later acceleration and velocity peaks were found on X than on T (*t* = 2.11; *p* = 0.030 and *t* = 3.78; *p* = 0.0024, respectively). The velocity peak furthermore occurred earlier on Z than on X (*t* = −3.69; *p* = 0.004). **(B)** For congruent verbal instructions, effects were only seen for +45° targets: acceleration peaked later on X than on T (*t* = 2.15; *p* = 0.045) and Z (*t* = −2.69; *p* = 0.020). The velocity peak was found to occur earlier on Z than on both X (*t* = −3.12; *p* = 0.009) and T (*t* = −3.46; *p* = 0.004).

Supplementary Figure 2 Temporal dynamic of the movements toward −45° and +45° targets upon **(A)** visual and **(B)** congruent verbal instructions, for the acceleration (left panel) and velocity (right panel) peaks in Experiment 2. Latencies (in milliseconds) of the peaks along the horizontal (X), tangential (T) and vertical (Z) axes are reported (see Supplementary Figure 1 for conventions; * indicates a significant difference between conditions). **(A)** In the visual block, for −45° targets, the acceleration peak occurred later on Z than on X (*t* = 5.03; *p* = 0.0008) and T (*t* = 3.63; *p* = 0.0004). For +45° targets, acceleration and velocity peaked later on X than on T (acceleration: *t* = 2.72; *p* = 0.01; velocity: *t* = 3.19; *p* = 0.0004, respectively) and Z (acceleration: *t* = −2.65; *p* = 0.015; velocity: *t* = −2.75; *p* = 0.005). **(B)** For congruent verbal instructions, differences between conditions were only found for +45° targets: acceleration and velocity peaked later on X than on T (acceleration: *t* = 3.58; *p* = 0.0032; velocity: *t* = 3.88; *p* = 0.0004) and Z (acceleration: *t* = −3.84; *p* = 0.0016; velocity: *t* = −4.58; *p* = 0.0008). In addition, the velocity peak occurred earlier on Z than on T (*t* = −3.10; *p* = 0.003).

Supplementary Figure 3 Effect of verbal incongruency on the temporal dynamic of movements toward −45° (left panel) and +45° (right panel) targets in Experiment 2. Latencies (in milliseconds) of the acceleration (top panel) and velocity (bottom panel) peaks extracted from the horizontal (X), tangential (T) and vertical (Z) axes are reported (see Supplementary Figure 2 for conventions; * indicates a significant difference between conditions). Left panel: for movements toward −45° targets following incongruent verbal instructions, both acceleration and velocity peaked earlier on Z than on T and X (for T: acceleration: *t* = 3.85; *p* = 0.0008; velocity: *t* = 3.30; *p* = 0.0016; for X: acceleration: *t* = 3.70; *p* = 0.002; velocity: *t* = 2.87; *p* = 0.0036). On the other hand, the acceleration peak occurred earlier on X than on T (*t* = −2.54; *p* = 0.023). Right panel: for movements toward +45° targets, the acceleration peak showed longer latency on Z with respect to T (*t* = 3.85; *p* = 0.0008) but the Fischer combination test did not reach significance (T vs. Z: *K* = 5.7; *p* = 0.1).

## References

[B1] Aravena P. Delevoye-Turrell Y. Deprez V. Cheylus A. Paulignan Y. Frak V. (2012). Grip force reveals the context sensitivity of language-induced motor activity during “action words” processing : Evidence from sentential negation. *PLoS One* 7:e50287. 10.1371/journal.pone.0050287 23227164 PMC3515598

[B2] Arbib M. A. (2002). Beyond the mirror system: From monkey-like action recognition to human language. *Behav. Brain Sci.* 28 105–124. 10.1017/s0140525x05000038 16201457

[B3] Bardolph M. Coulson S. (2014). How vertical hand movements impact brain activity elicited by literally and metaphorically related words: An ERP study of embodied metaphor. *Front. Hum. Neurosci.* 8:1031. 10.3389/fnhum.2014.01031 25566041 PMC4274969

[B4] Basso D. Finos L. (2012). Exact multivariate permutation tests for fixed effects in mixed-models. *Commun. Stat. Theory Methods* 41 2991–3001. 10.1080/03610926.2011.627103

[B5] Bates E. Dick F. (2002). Language, gesture, and the developing brain. *Dev. Psychobiol.* 40 293–310. 10.1002/dev.10034 11891640

[B6] Bernardis P. Bello A. Pettenati P. Stefanini S. Gentilucci M. (2008). Manual actions affect vocalizations of infants. *Exp. Brain Res.* 184 599–603. 10.1007/s00221-007-1256-x 18183374

[B7] Berret B. Darlot C. Jean F. Pozzo T. Papaxanthis C. Gauthier J. P. (2008). The Inactivation Principle: Mathematical solutions minimizing the absolute work and biological implications for the planning of arm movements. *PLoS Comput. Biol.* 4:e1000194. 10.1371/journal.pcbi.1000194 18949023 PMC2561290

[B8] Boersma P. Weenik D. (2012). *Doing phonetics by computer [Computer program].* Available online at: http://www.praat.org/ (accessed June 30, 2020).

[B9] Borreggine K. L. Kaschak M. P. (2006). The action–sentence compatibility effect: It’s all in the timing. *Cogn. Sci.* 30 1097–1112. 10.1207/s15516709cog0000_91 21702848

[B10] Boulenger V. Hauk O. Pulvermüller F. (2009). Grasping ideas with the motor system: Semantic somatotopy in idiom comprehension. *Cereb. Cortex* 19 1905–1914. 10.1093/cercor/bhn217 19068489 PMC2705699

[B11] Boulenger V. Martel M. Bouvet C. Finos L. Krzonowski J. Farnè A. (2020). Feeling better: Tactile verbs speed up tactile detection. *Brain Cogn.* 142:105582. 10.1016/j.bandc.2020.105582 32422452

[B12] Boulenger V. Roy A. C. Paulignan Y. Deprez V. Jeannerod M. Nazir T. A. (2006). Cross-talk between language processes and overt motor behavior in the first 200 msec of processing. *J. Cogn. Neurosci.* 18 1607–1615. 10.1162/jocn.2006.18.10.1607 17014366

[B13] Boulenger V. Silber B. Y. Roy A. C. Paulignan Y. Jeannerod M. Nazir T. A. (2008). Subliminal display of action words interferes with motor planning: A combined EEG and kinematic study. *J. Physiol. Paris* 102 130–136. 10.1016/j.jphysparis.2008.03.015 18485678

[B14] Brookshire G. Casasanto D. Ivry R. (2010). “Modulation of motor-meaning congruity effects for valenced words,” in *Proceedings of the 32nd Annual Meeting of the Cognitive Science Society (CogSci 2010)*, eds Ohlsson S. Catrambone R. (Austin, TX: Cognitive Science Society), 1940–1945.

[B15] Brouwer H. Fitz H. Hoeks J. (2012). Getting real about semantic illusions: Rethinking the functional role of the P600 in language comprehension. *Brain Res.* 1446 127–143. 10.1016/j.brainres.2012.01.055 22361114

[B16] Brozzoli C. Roy A. C. Lidborg L. H. Lövdén M. (2019). Language as a tool: Motor proficiency using a tool predicts individual linguistic abilities. *Fronti. Psychol.* 10:1639. 10.3389/fpsyg.2019.01639 31379674 PMC6659550

[B17] Bryant D. J. Tversky B. Franklin N. (1992). Internal and external spatial frameworks for representing described scenes. *J. Mem. Lang.* 31 74–98. 10.1016/0749-596X(92)90006-J

[B18] Casasanto D. Dijkstra K. (2010). Motor action and emotional memory. *Cognition* 115 179–185. 10.1016/j.cognition.2009.11.002 20096831 PMC2830276

[B19] Cian L. (2017). Verticality and conceptual metaphors: A systematic review. *J. Assoc. Consum. Res.* 2 444–459. 10.1086/694082

[B20] CLARK N. Perlman M. Johansson Falck M. (2013a). “Iconic pitch expresses vertical space,” in *Language and the creative mind*, eds Borkent M. Dancygier B. Hinnell J. (Stanford, CA: CSLI Publications), 393–410.

[B21] Connell L. Cai Z. G. Holler J. (2013). Do you see what I’m singing? Visuospatial movement biases pitch perception. *Brain Cogn.* 81 124–130. 10.1016/j.bandc.2012.09.005 23195703

[B22] Corballis M. C. (2002). *From hand to mouth: The origins of language.* Princeton: Princeton University Press.

[B23] Corballis M. C. (2010). Mirror neurons and the evolution of language. *Brain Lang.* 112 25–35. 10.1016/j.bandl.2009.02.002 19342089

[B24] Crevecoeur F. Thonnard J.-L. Lefèvre P. (2009). Optimal integration of gravity in trajectory planning of vertical pointing movements. *J. Neurophysiol.* 102 786–796. 10.1152/jn.00113.2009 19458149

[B25] D’Ausilio A. Craighero L. Fadiga L. (2012). The contribution of the frontal lobe to the perception of speech. *J. Neurolinguistics* 25 328–335. 10.1016/j.jneuroling.2010.02.003

[B26] Desai R. H. Binder J. R. Conant L. L. Seidenberg M. S. (2010). Activation of sensory-motor areas in sentence comprehension. *Cereb. Cortex* 20 468–478. 10.1093/cercor/bhp115 19546154 PMC2803740

[B27] Dolscheid S. Hunnius S. Casasanto D. Majid A. (2014). Prelinguistic infants are sensitive to space-pitch associations found across cultures. *Psychol. Sci.* 25 1256–1261. 10.1177/0956797614528521 24899170

[B28] Dolscheid S. Shayan S. Majid A. Casasanto D. (2013). The thickness of musical pitch: Psychophysical evidence for linguistic relativity. *Psychol. Sci.* 24 613–621. 10.1177/0956797612457374 23538914

[B29] Dudschig C. de la Vega I. Kaup B. (2015). What’s up? Emotion-specific activation of vertical space during language processing. *Acta Psychol.* 156 143–155. 10.1016/j.actpsy.2014.09.015 25454886

[B30] Eitan Z. Timmers R. (2010). Beethoven’s last piano sonata and those who follow crocodiles: Cross-domain mappings of auditory pitch in a musical context. *Cognition* 114 405–422. 10.1016/j.cognition.2009.10.013 20036356

[B31] Fargier R. Ménoret M. Boulenger V. Nazir T. A. Paulignan Y. (2012). Grasp it loudly! Supporting actions with semantically congruent spoken action words. *PLoS One* 7:e30663. 10.1371/journal.pone.0030663 22292014 PMC3265503

[B32] Farnè A. Roy A. C. Giraux P. Dubernard J. M. Sirigu A. (2002). Face or hand, not both: Perceptual correlates of reafferentation in a former amputee. *Curr. Biol.* 12 1342–1346. 10.1016/s0960-9822(02)01018-7 12176365

[B33] Fernandez-Prieto I. Spence C. Pons F. Navarra J. (2017). Does language influence the vertical representation of auditory pitch and loudness? *Iperception* 8:2041669517716183. 10.1177/2041669517716183 28694959 PMC5484432

[B34] Ferragne E. Flavier S. Fressard C. (2012). *ROCme! Recording of oral corpora made easy (2.0) [Computer software].* Available online at: www.ddl.cnrs.fr/rocme (accessed June 15, 2020).

[B35] Finos L. Basso D. (2014). Permutation tests for between-unit fixed effects in multivariate generalized linear mixed models. *Stat. Comput.* 24 941–952. 10.1007/s11222-013-9412-6

[B36] Fischer M. H. Zwaan R. A. (2008). Embodied language: A review of the role of the motor system in language comprehension. *Q. J. Exp. Psychol.* 61 825–850. 10.1080/17470210701623605 18470815

[B37] Flöel A. Ellger T. Breitenstein C. Knecht S. (2003). Language perception activates the hand motor cortex: Implications for motor theories of speech perception. *Eur. J. Neurosci.* 18 704–708. 10.1046/j.1460-9568.2003.02774.x 12911767

[B38] Forker D. (2020). Elevation as a grammatical and semantic category of demonstratives. *Front. Psychol.* 11:1712. 10.3389/fpsyg.2020.01712 32849028 PMC7406794

[B39] Frak V. Nazir T. Goyette M. Cohen H. Jeannerod M. (2010). Grip force is part of the semantic representation of manual action verbs. *PLoS One* 5:e9728. 10.1371/journal.pone.0009728 20300535 PMC2838801

[B40] Gallese V. Lakoff G. (2005). The brain’s concepts: The role of the sensory-motor system in conceptual knowledge. *Cogn. Neuropsychol.* 22 455–479. 10.1080/02643290442000310 21038261

[B41] Gaveau J. Papaxanthis C. (2011). The temporal structure of vertical arm movements. *PLoS One* 6:e22045. 10.1371/journal.pone.0022045 21765935 PMC3134452

[B42] Gaveau J. Berret B. Angelaki D. E. Papaxanthis C. (2016). Direction-dependent arm kinematics reveal optimal integration of gravity cues. *eLife* 5:e16394. 10.7554/eLife.16394 27805566 PMC5117856

[B43] Gaveau J. Grospretre S. Berret B. Angelaki D. E. Papaxanthis C. (2021). A cross-species neural integration of gravity for motor optimization. *Sci. Adv.* 7:eabf7800. 10.1126/sciadv.abf7800 33827823 PMC8026131

[B44] Gentili R. Cahouet V. Papaxanthis C. (2007). Motor planning of arm movements is direction-dependent in the gravity field. *Neuroscience* 145 20–32. 10.1016/j.neuroscience.2006.11.035 17224242

[B45] Gentilucci M. (2003). Grasp observation influences speech production. *Eur. J. Neurosci.* 17 179–184. 10.1046/j.1460-9568.2003.02438.x 12534983

[B46] Gentilucci M. Campione G. C. (2011). Do postures of distal effectors affect the control of actions of other distal effectors? Evidence for a system of interactions between hand and mouth. *PLoS One* 6:e19793. 10.1371/journal.pone.0019793 21625428 PMC3100300

[B47] Gentilucci M. Corballis M. (2006). From manual gesture to speech: A gradual transition. *Neurosci. Biobehav. Rev.* 30 949–960. 10.1016/j.neubiorev.2006.02.004 16620983

[B48] Gentilucci M. Gangitano M. (1998). Influence of automatic word reading on motor control. *Eur. J. Neurosci.* 10 752–756. 10.1046/j.1460-9568.1998.00060.x 9749737

[B49] Gentilucci M. Benuzzi F. Bertolani L. Daprati E. Gangitano M. (2000). Language and motor control. *Exp. Brain Res.* 133 468–490. 10.1007/s002210000431 10985682

[B50] Gentilucci M. Campione G. C. Dalla Volta R. Bernardis P. (2009). The observation of manual grasp actions affects the control of speech: A combined behavioral and transcranial magnetic stimulation study. *Neuropsychologia* 47 3190–3202. 10.1016/j.neuropsychologia.2009.07.020 19654016

[B51] Gentilucci M. Santunione P. Roy A. C. Stefanini S. (2004a). Execution and observation of bringing a fruit to the mouth affect syllable pronunciation. *Eur. J. Neurosci.* 19 190–202. 10.1111/j.1460-9568.2004.03104.x 14750977

[B52] Gentilucci M. Stefanini S. Roy A. C. Santunione P. (2004b). Action observation and speech production: Study on children and adults. *Neuropsychologia* 42 1554–1567. 10.1016/j.neuropsychologia.2004.03.002 15246292

[B53] Gianelli C. Farnè A. Salemme R. Jeannerod M. Roy A. C. (2011). The agent is right: When motor embodied cognition is space-dependent. *PLoS One* 6:e25036. 10.1371/journal.pone.0025036 21966407 PMC3179480

[B54] Glenberg A. M. Kaschak M. P. (2002). Grounding language in action. *Psychon. Bull. Rev.* 9 558–565. 12412897 10.3758/bf03196313

[B55] Globig L. K. Hartmann M. Martarelli C. S. (2019). Vertical head movements influence memory performance for words with emotional content. *Front. Psychol.* 10:672. 10.3389/fpsyg.2019.00672 30971992 PMC6443899

[B56] Gonzalez S. L. Alvarez V. Nelson E. L. (2019). Do gross and fine motor skills differentially contribute to language outcomes? A systematic review. *Front. Psychol.* 10:2670. 10.3389/fpsyg.2019.02670 31849775 PMC6901663

[B57] Graham J. A. Heywood S. (1975). The effects of elimination of hand gestures and of verbal codability on speech performance. *Eur. J. Soc. Psychol.* 5 189–195. 10.1002/ejsp.2420050204

[B58] Hadar U. Wenkert-Olenik D. Krauss R. Soroker N. (1998). Gesture and the processing of speech: Neuropsychological evidence. *Brain Lang.* 62 107–126. 10.1006/brln.1997.1890 9570882

[B59] Hauk O. Pulvermüller F. (2004). Neurophysiological distinction of action words in the fronto-central cortex: Neurophysiological distinction of action words. *Hum. Brain Mapp.* 21 191–201. 10.1002/hbm.10157 14755838 PMC6872027

[B60] Hauk O. Johnsrude I. Pulvermüller F. (2004). Somatotopic representation of action words in human motor and premotor cortex. *Neuron* 41 301–307. 14741110 10.1016/s0896-6273(03)00838-9

[B61] Higginbotham D. R. Isaak M. I. Domingue J. N. (2008). The exaptation of manual dexterity for articulate speech: An electromyogram investigation. *Exp. Brain Res.* 186 603–609. 10.1007/s00221-007-1265-9 18224308

[B62] Hill E. L. (2001). Non-specific nature of specific language impairment: A review of the literature with regard to concomitant motor impairments. *Int. J. Lang. Commun. Disord.* 36 149–171. 10.1080/13682820010019874 11344592

[B63] Holler J. Drijvers L. Rafiee A. Majid A. (2022). Embodied space-pitch associations are shaped by language. *Cogn. Sci.* 46:e13083. 10.1111/cogs.13083 35188682

[B64] Imbert C. (2012). Path: Ways typology has walked through it. *Lang Ling Compass* 6 236–258.

[B65] Imbert C. (2013). Morpheme order constraints upside down: Verticality and other directions. *Annu. Meet. Berkeley Linguist. Soc.* 39:396. 10.3765/bls.v39i1.3895 26464298

[B66] Imbert C. (2016). “Morpheme order constraints upside down: Verticality and other directions,” in *Proceedings of the annual meeting of the berkeley linguistics society*, Berkeley, CA, 39.

[B67] Iverson J. M. (2010). Developing language in a developing body: The relationship between motor development and language development. *J. Child Lang.* 37 229–261. 10.1017/S0305000909990432 20096145 PMC2833284

[B68] Iverson J. M. Braddock B. (2011). Gesture and motor skill in relation to language in children with language impairment. *J. Speech Lang. Hear. Res.* 54 72–86. 10.1044/1092-4388(2010/08-0197 20719867

[B69] Iverson J. M. Goldin-Meadow S. (1998). Why people gesture when they speak. *Nature* 396 228–228. 10.1038/24300 9834030

[B70] Iverson J. M. Goldin-Meadow S. (2005). Gesture paves the way for language development. *Psychol. Sci.* 16 367–371. 10.1111/j.0956-7976.2005.01542.x 15869695

[B71] Iverson J. M. Capirci O. Volterra V. Goldin-Meadow S. (2008). Learning to talk in a gesture-rich world: Early communication in Italian vs. American children. *First Lang.* 28 164–181. 10.1177/0142723707087736 19763226 PMC2744975

[B72] Iverson J. M. Hall A. J. Nickel L. Wozniak R. H. (2007). The relationship between reduplicated babble onset and laterality biases in infant rhythmic arm movements. *Brain Lang.* 101 198–207. 10.1016/j.bandl.2006.11.004 17196644 PMC2034511

[B73] Jiang M. Henley T. (2012). Power and spatial relations. *J. Cogn. Psychol.* 24 829–835. 10.1080/20445911.2012.702749

[B74] Kaschak M. P. Borreggine K. L. (2008). Temporal dynamics of the action–sentence compatibility effect. *Q. J. Exp. Psychol.* 61 883–895. 10.1080/17470210701623852 18470819 PMC4612619

[B75] Kaschak M. P. Madden C. J. Therriault D. J. Yaxley R. H. Aveyard M. Blanchard A. A. (2005). Perception of motion affects language processing. *Cognition* 94 B79–B89. 10.1016/j.cognition.2004.06.005 15617669

[B76] Kövecses Z. (2008). “Universality and variation in the use of metaphor,” in *Selected paper, from the 2006 and 2007 stockholm metaphor festivals*, eds Johannesson N. L. Minugh D. C. (Stockholm, SE: Stockholm University), 51–74.

[B77] Krauss R. Chen Y. Gottesman R. (2000). “Lexical gestures and lexical access: A process model,” in *Language and gesture: Window into thought and action*, ed. McNeill D. (Cambridge: Cambridge University Press), 228–261.

[B78] Lachmair M. Fernandez S. R. Bury N.-A. Gerjets P. Fischer M. H. Bock O. L. (2016). How body orientation affects concepts of space, time and valence: Functional relevance of integrating sensorimotor experiences during word processing. *PLoS One* 11:e0165795. 10.1371/journal.pone.0165795 27812155 PMC5094761

[B79] Lakoff G. Johnson M. (2003). *Metaphors we live by*, 1st Edn. Chicago: University of Chicago Press.

[B80] Lakoff G. Johnson M. L. (1999). *Philosophy in the flesh: The embodied mind and its challenge to western thought.* New York, NY: Basic Books. 10.5860/choice.37-0239

[B81] Lanyon L. Rose M. L. (2009). Do the hands have? it The facilitation effects of arm and hand gesture on word retrieval in aphasia. *Aphasiology* 23 809–822. 10.1080/02687030802642044

[B82] Le Seac’h A. B. McIntyre J. (2007). Multimodal reference frame for the planning of vertical arms movements. *Neurosci. Lett.* 423 211–215. 10.1016/j.neulet.2007.07.034 17709199

[B83] Levinson S. C. (2003). *Space in language and cognition: Explorations in cognitive diversity.* Cambridge: Cambridge University Press.

[B84] Locke J. L. Bekken K. E. McMinn-Larson L. Wein D. (1995). Emergent control of manual and vocal-motor activity in relation to the development of speech. *Brain Lang.* 51 498–508. 10.1006/brln.1995.1073 8719079

[B85] Łozińska J. (2018). *Path and manner saliency in polish in contrast with Russian: A cognitive linguistic study.* Leiden: Brill.

[B86] Łozińska J. (2021). The poverty of manner categories in motion verbs coding vertical relations. Evidence from polish and Russian. *Russ. Linguist.* 45 93–104. 10.1007/s11185-021-09237-2

[B87] Łozińska J. Pietrewicz B. (2018). Lexicalisation of vertical motion: A study of three satellite-framed languages. *Cogn. Stud. Études Cogn.* 18:18. 10.11649/cs.1601

[B88] Maeda F. Kanai R. Shimojo S. (2004). Changing pitch induced visual motion illusion. *Curr. Biol.* 14 R990–R991. 10.1016/j.cub.2004.11.018 15589145

[B89] Martel M. Fourneret P. Finos L. Schmitz C. Catherine Roy A. (2020). Highs and lows in motor control development. *J. Motor Behav.* 52 404–417. 10.1080/00222895.2019.1643283 31339466

[B90] Meier B. P. Robinson M. D. (2004). Why the sunny side is up: Associations between affect and vertical position. *Psychol. Sci.* 15 243–247. 10.1111/j.0956-7976.2004.00659.x 15043641

[B91] Meister I. G. Wilson S. M. Deblieck C. Wu A. D. Iacoboni M. (2007). The essential role of premotor cortex in speech perception. *Curr. Biol.* 17 1692–1696. 10.1016/j.cub.2007.08.064 17900904 PMC5536895

[B92] Melinger A. Levelt W. J. M. (2004). Gesture and the communicative intention of the speaker. *Gesture* 4 119–141. 10.1075/gest.4.2.02mel 33486653

[B93] Merriaux P. Dupuis Y. Boutteau R. Vasseur P. Savatier X. (2017). A study of vicon system positioning performance. *Sensors* 17:1591. 10.3390/s17071591 28686213 PMC5551098

[B94] Meteyard L. Bahrami B. Vigliocco G. (2007). Motion detection and motion verbs language affects low-level visual perception. *Psychol. Sci.* 18 1007–1013. 10.1111/j.1467-9280.2007.02016.x 17958716

[B95] Meteyard L. Zokaei N. Bahrami B. Vigliocco G. (2008). Visual motion interferes with lexical decision on motion words. *Curr. Biol.* 18 R732–R733. 10.1016/j.cub.2008.07.016 18786369

[B96] Morett L. M. Chang L.-Y. (2015). Emphasising sound and meaning: Pitch gestures enhance Mandarin lexical tone acquisition. *Lang. Cogn. Neurosci.* 30 347–353. 10.1080/23273798.2014.923105

[B97] Morett L. M. Feiler J. B. Getz L. M. (2022). Elucidating the influences of embodiment and conceptual metaphor on lexical and non-speech tone learning. *Cognition* 222:105014. 10.1016/j.cognition.2022.105014 35033864

[B98] Naigles L. R. Eisenberg A. R. Kako E. T. Highter M. McGraw N. (1998). Speaking of motion: Verb use in english and spanish. *Lang. Cogn. Process.* 13 521–549. 10.1080/016909698386429

[B99] Nazir T. A. Boulenger V. Roy A. Silber B. Jeannerod M. Paulignan Y. (2008). Language-induced motor perturbations during the execution of a reaching movement. *Q. J. Exp. Psychol.* 61 933–943. 10.1080/17470210701625667 18470823

[B100] Oldfield R. C. (1971). The assessment and analysis of handedness: The Edinburgh inventory. *Neuropsychologia* 9 97–113. 10.1016/0028-3932(71)90067-4 5146491

[B101] Oosterwijk S. Rotteveel M. Fischer A. H. Hess U. (2009). Embodied emotion concepts: How generating words about pride and disappointment influences posture. *Eur. J. Soc. Psychol.* 39 457–466. 10.1002/ejsp.584

[B102] Papaxanthis C. Dubost V. Pozzo T. (2003). Similar planning strategies for whole-body and arm movements performed in the sagittal plane. *Neuroscience* 117 779–783. 10.1016/s0306-4522(02)00964-8 12654330

[B103] Papaxanthis C. Pozzo T. McIntyre J. (2005). Kinematic and dynamic processes for the control of pointing movements in humans revealed by short-term exposure to microgravity. *Neuroscience* 135 371–383. 10.1016/j.neuroscience.2005.06.063 16125854

[B104] Papaxanthis C. Pozzo T. Stapley P. (1998). Effects of movement direction upon kinematic characteristics of vertical arm pointing movements in man. *Neurosci. Lett.* 253 103–106. 10.1016/S0304-3940(98)00604-1 9774160

[B105] Pesarin F. (2001). *Multivariate permutation tests: With applications in biostatistics. Undefined.* Available online at: https://www.semanticscholar.org/paper/Multivariate-Permutation-Tests-%3A-With-Applications-Pesarin/7ef57d33dd28cbccc7eeed735fa1a4400e98b8be (accessed May 20, 2002).

[B106] Poirier G. Papaxanthis C. Mourey F. Gaveau J. (2020). Motor planning of vertical arm movements in healthy older adults: Does effort minimization persist with aging? *Front. Aging Neurosci.* 12:37. 10.3389/fnagi.2020.00037 32161533 PMC7052522

[B107] Pulvermüller F. Fadiga L. (2010). Active perception: Sensorimotor circuits as a cortical basis for language. *Nat. Rev. Neurosci.* 11 351–360. 10.1038/nrn2811 20383203

[B108] Pulvermüller F. Lutzenberger W. Preissl H. (1999). Nouns and verbs in the intact brain: Evidence from event-related potentials and high-frequency cortical responses. *Cereb. Cortex* 9 497–506. 10.1093/cercor/9.5.497 10450894

[B109] Richardson D. C. Spivey M. J. Barsalou L. W. McRae K. (2003). Spatial representations activated during real-time comprehension of verbs. *Cogn. Sci.* 27 767–780. 10.1207/s15516709cog2705_4

[B110] Roy A. C. Craighero L. Fabbri-Destro M. Fadiga L. (2008). Phonological and lexical motor facilitation during speech listening: A transcranial magnetic stimulation study. *J. Physiol. Paris* 102 101–105. 10.1016/j.jphysparis.2008.03.006 18440210

[B111] Roy A. C. Curie A. Nazir T. Paulignan Y. des Portes V. Fourneret P. (2013). Syntax at hand: Common syntactic structures for actions and language. *PLoS One* 8:e72677. 10.1371/journal.pone.0072677 23991140 PMC3749983

[B112] Rusconi E. Kwan B. Giordano B. L. Umiltà C. Butterworth B. (2006). Spatial representation of pitch height: The SMARC effect. *Cognition* 99 113–129. 10.1016/j.cognition.2005.01.004 15925355

[B113] Santana E. de Vega M. (2011). Metaphors are embodied, and so are their literal counterparts. *Front. Psychol.* 2:90. 10.3389/fpsyg.2011.00090 21687459 PMC3110336

[B114] Sasaki K. Yamada Y. Miura K. (2016). Emotion biases voluntary vertical action only with visible cues. *Acta Psychol.* 163 97–106. 10.1016/j.actpsy.2015.11.003 26637931

[B115] Schubert T. W. (2005). Your highness: Vertical positions as perceptual symbols of power. *J. Pers. Soc. Psychol.* 89 1–21. 10.1037/0022-3514.89.1.1 16060739

[B116] Senot P. Zago M. Lacquaniti F. McIntyre J. (2005). Anticipating the effects of gravity when intercepting moving objects: Differentiating up and down based on nonvisual cues. *J. Neurophysiol.* 94 4471–4480. 10.1152/jn.00527.2005 16120661

[B117] Shayan S. Ozturk O. Sicoli M. (2011). The thickness of pitch: Crossmodal metaphors in Farsi, Turkish, and Zapotec. *Senses Soc.* 6 96–105. 10.2752/174589311X12893982233911

[B118] Talmy L. (1985). “Lexicalization patterns: Semantic structure in lexical form,” in *Language typology and syntactic description*, Vol. 3 ed. Shopen T. (Amsterdam: John Benjamins), 57–149.

[B119] Talmy L. (1991). “Path to realization: A typology of event conflation,” in *Proceedings of the berkeley linguistics society*, Vol. 17 Berkeley, CA, 480–520.

[B120] Talmy L. (2000). *Toward a cognitive semantics: Concept structuring systems*, Vol. 1. Bradford: Bradford Book.

[B121] Tettamanti M. Buccino G. Saccuman M. C. Gallese V. Danna M. Scifo P. (2005). Listening to action-related sentences activates fronto-parietal motor circuits. *J. Cogn. Neurosci.* 17 273–281. 10.1162/0898929053124965 15811239

[B122] Thibault S. Py R. Gervasi A. M. Salemme R. Koun E. Lövden M. (2021). Tool use and language share syntactic processes and neural patterns in the basal ganglia. *Science* 374:eabe0874. 10.1126/science.abe0874 34762470

[B123] Tiainen M. Tiippana K. Vainio M. Komeilipoor N. Vainio L. (2017). Interaction in planning vocalizations and grasping. *Q. J. Exp. Psychol.* 70 1590–1602. 10.1080/17470218.2016.1195416 27251752

[B124] Troje N. F. Westhoff C. (2006). The inversion effect in biological motion perception: Evidence for a ≪ life detector ≫? *Curr. Biol.* 16 821–824. 10.1016/j.cub.2006.03.022 16631591

[B125] Vainio L. Rantala A. Tiainen M. Tiippana K. Komeilipoor N. Vainio M. (2017). Systematic influence of perceived grasp shape on speech production. *PLoS One* 12:e0170221. 10.1371/journal.pone.0170221 28103278 PMC5245887

[B126] Vainio L. Schulman M. Tiippana K. Vainio M. (2013). Effect of syllable articulation on precision and power grip performance. *PLoS One* 8:e53061. 10.1371/journal.pone.0053061 23326381 PMC3541367

[B127] Vainio L. Tiainen M. Tiippana K. Vainio M. (2014). Shared processing of planning articulatory gestures and grasping. *Exp. Brain Res.* 232 2359–2368. 10.1007/s00221-014-3932-y 24710666

[B128] Walker P. Bremner J. G. Mason U. Spring J. Mattock K. Slater A. (2010). Preverbal infants’ sensitivity to synaesthetic cross-modality correspondences. *Psychol. Sci.* 21 21–25. 10.1177/0956797609354734 20424017

[B129] Wang Y. Zhang X. Wang C. Huang W. Xu Q. Liu D. (2022). Modulation of biological motion perception in humans by gravity. *Nat. Commun.* 13:2765. 10.1038/s41467-022-30347-y 35589705 PMC9120521

[B130] White O. Gaveau J. Bringoux L. Crevecoeur F. (2020). The gravitational imprint on sensorimotor planning and control. *J. Neurophysiol.* 124 4–19. 10.1152/jn.00381.2019 32348686

[B131] Willems R. M. Hagoort P. (2007). Neural evidence for the interplay between language, gesture, and action: A review. *Brain Lang.* 101 278–289. 10.1016/j.bandl.2007.03.004 17416411

[B132] Windolf M. Götzen N. Morlock M. (2008). Systematic accuracy and precision analysis of video motion capturing systems—Exemplified on the Vicon-460 system. *J. Biomech.* 41 2776–2780. 10.1016/j.jbiomech.2008.06.024 18672241

[B133] Wnuk E. Ito Y. (2021). The heart’s downward path to happiness: Cross-cultural diversity in spatial metaphors of affect. *Cogn. Linguist.* 32 195–218. 10.1515/cog-2020-0068

[B134] Yamamoto S. Fujii K. Zippo K. Kushiro K. Araki M. (2019). The kinetic mechanisms of vertical pointing movements. *Heliyon* 5:e02012. 10.1016/j.heliyon.2019.e02012 31360781 PMC6637177

[B135] Zwaan R. A. Taylor L. J. (2006). Seeing, acting, understanding: Motor resonance in language comprehension. *J. Exp. Psychol. Gen.* 135 1–11. 16478313 10.1037/0096-3445.135.1.1

[B136] Zwaan R. A. Yaxley R. H. (2003). Spatial iconicity affects semantic relatedness judgments. *Psychon. Bull. Rev.* 10 954–958. 10.3758/BF03196557 15000544

[B137] Zwaan R. A. Madden C. J. Yaxley R. H. Aveyard M. E. (2004). Moving words: Dynamic representations in language comprehension*. *Cogn. Sci.* 28 611–619. 10.1207/s15516709cog2804_5 33486653

