# Supplementary figures and images for: Up right, not right up: Primacy of verticality in both language and movement
Source: Front Hum Neurosci. 2022 Sep 29;16:981330. doi: 10.3389/fnhum.2022.981330 (PMC9558293; doi:10.3389/fnhum.2022.981330)

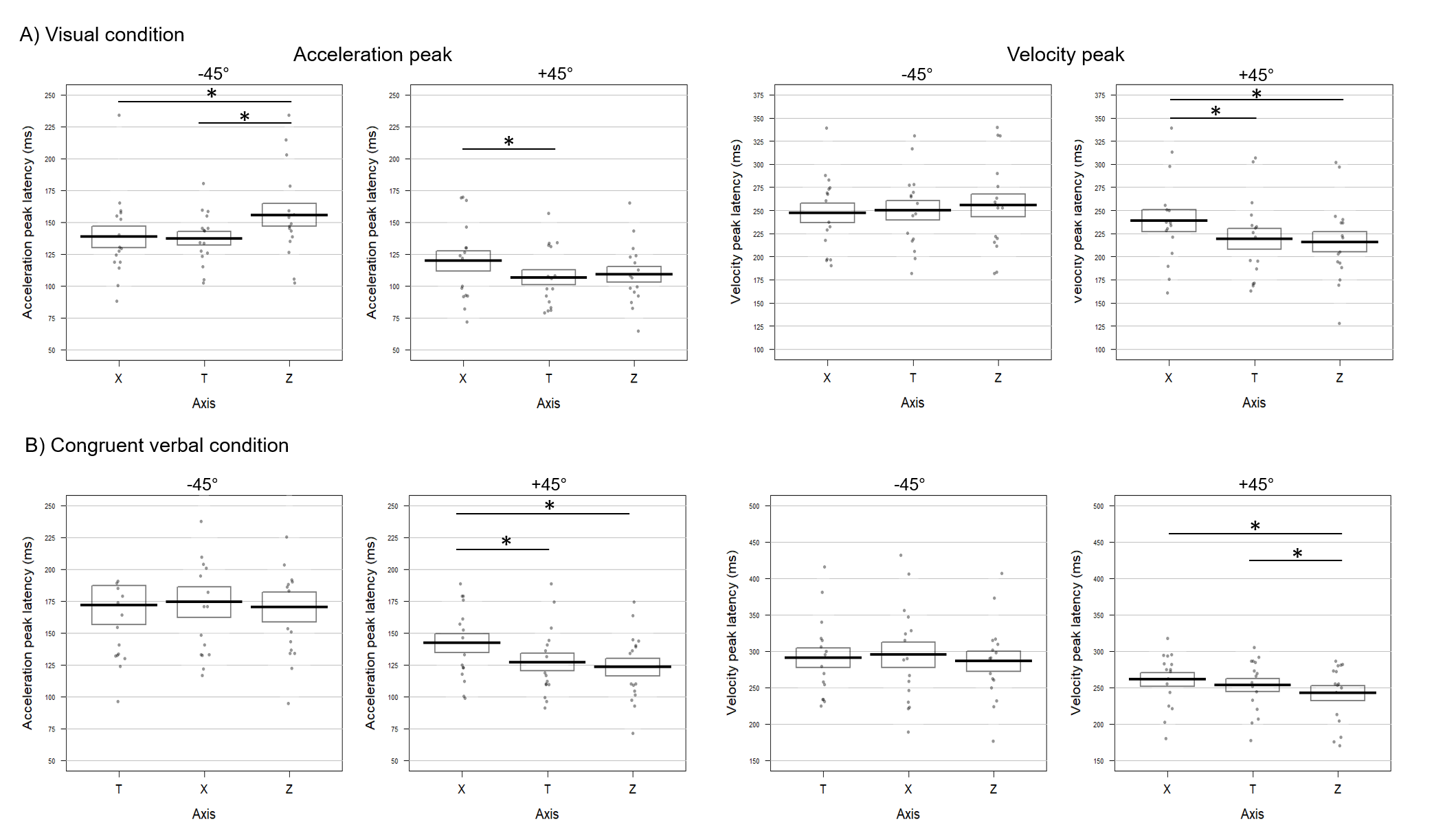

Supplement: Supplementary Figure 1 — Temporal dynamic of the movements toward −45° and +45° targets upon (A) visual and (B) congruent verbal instructions, for the acceleration (left panel) and velocity (right panel) peaks in Experiment 1. Latencies (in milliseconds) of the peaks along the horizontal (X), tangential (T), and vertical (Z) axes are reported. The bold lines represent peak latencies averaged across participants for each axis, rectangles illustrate the standard errors. Each dot stands for the mean peak latency for one participant in the corresponding condition. Asterisk indicates a significant difference between the conditions. (A) Upon visual cues, when participants pointed toward −45° targets, acceleration peaked later on Z than on X (t = 3.10; p = 0.011) and T (t = 2.98; p = 0.014). For +45° targets, later acceleration and velocity peaks were found on X than on T (t = 2.11; p = 0.030 and t = 3.78; p = 0.0024, respectively). The velocity peak furthermore occurred earlier on Z than on X (t = −3.69; p = 0.004). (B) For congruent verbal instructions, effects were only seen for +45° targets: acceleration peaked later on X than on T (t = 2.15; p = 0.045) and Z (t = −2.69; p = 0.020). The velocity peak was found to occur earlier on Z than on both X (t = −3.12; p = 0.009) and T (t = −3.46; p = 0.004). [file Image_1.JPEG]

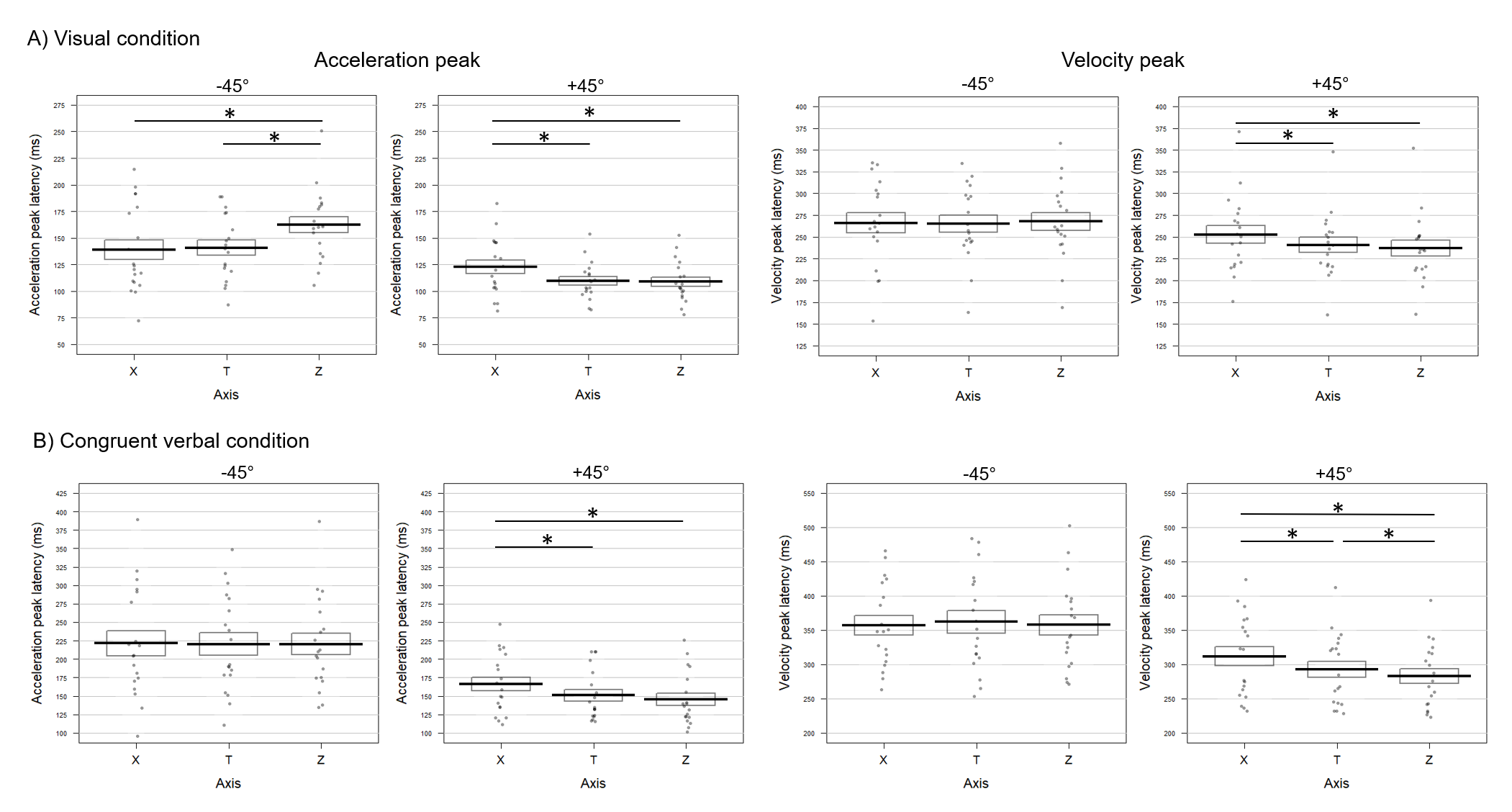

Supplement: Supplementary Figure 2 — Temporal dynamic of the movements toward −45° and +45° targets upon (A) visual and (B) congruent verbal instructions, for the acceleration (left panel) and velocity (right panel) peaks in Experiment 2. Latencies (in milliseconds) of the peaks along the horizontal (X), tangential (T) and vertical (Z) axes are reported (see Supplementary Figure 1 for conventions; * indicates a significant difference between conditions). (A) In the visual block, for −45° targets, the acceleration peak occurred later on Z than on X (t = 5.03; p = 0.0008) and T (t = 3.63; p = 0.0004). For +45° targets, acceleration and velocity peaked later on X than on T (acceleration: t = 2.72; p = 0.01; velocity: t = 3.19; p = 0.0004, respectively) and Z (acceleration: t = −2.65; p = 0.015; velocity: t = −2.75; p = 0.005). (B) For congruent verbal instructions, differences between conditions were only found for +45° targets: acceleration and velocity peaked later on X than on T (acceleration: t = 3.58; p = 0.0032; velocity: t = 3.88; p = 0.0004) and Z (acceleration: t = −3.84; p = 0.0016; velocity: t = −4.58; p = 0.0008). In addition, the velocity peak occurred earlier on Z than on T (t = −3.10; p = 0.003). [file Image_2.JPEG]

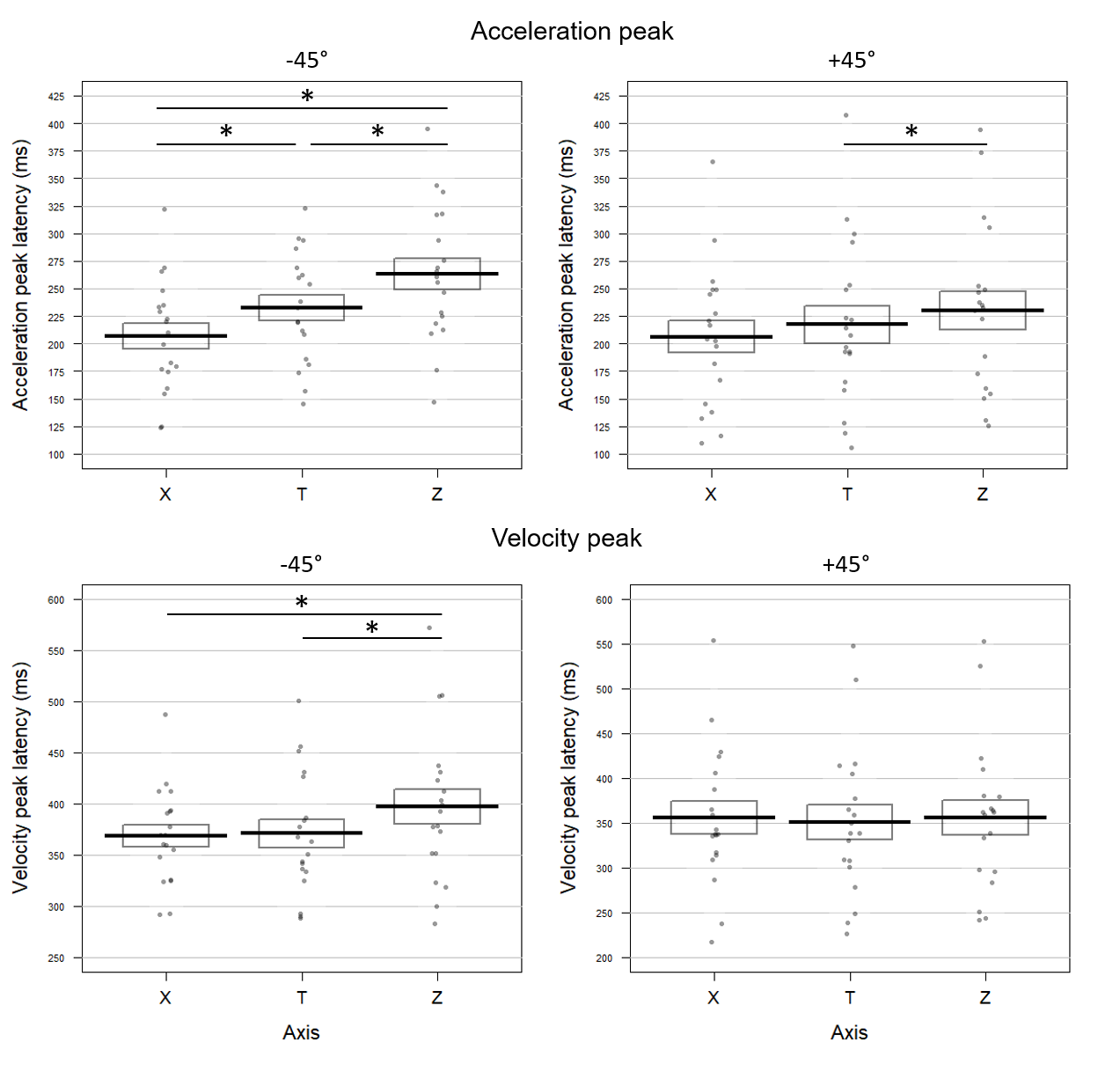

Supplement: Supplementary Figure 3 — Effect of verbal incongruency on the temporal dynamic of movements toward −45° (left panel) and +45° (right panel) targets in Experiment 2. Latencies (in milliseconds) of the acceleration (top panel) and velocity (bottom panel) peaks extracted from the horizontal (X), tangential (T) and vertical (Z) axes are reported (see Supplementary Figure 2 for conventions; * indicates a significant difference between conditions). Left panel: for movements toward −45° targets following incongruent verbal instructions, both acceleration and velocity peaked earlier on Z than on T and X (for T: acceleration: t = 3.85; p = 0.0008; velocity: t = 3.30; p = 0.0016; for X: acceleration: t = 3.70; p = 0.002; velocity: t = 2.87; p = 0.0036). On the other hand, the acceleration peak occurred earlier on X than on T (t = −2.54; p = 0.023). Right panel: for movements toward +45° targets, the acceleration peak showed longer latency on Z with respect to T (t = 3.85; p = 0.0008) but the Fischer combination test did not reach significance (T vs. Z: K = 5.7; p = 0.1). [file Image_3.JPEG]
